# Boosting immunity: synergistic antiviral effects of luteolin, vitamin C, magnesium and zinc against SARS-CoV-2 3CLpro

**DOI:** 10.1042/BSR20240617

**Published:** 2024-08-14

**Authors:** Juliana C. Ferreira, Samar Fadl, Thyago H.S. Cardoso, Bruno Silva Andrade, Tarcisio S. Melo, Edson Mario de Andrade Silva, Anupriya Agarwal, Stuart J. Turville, Nitin K. Saksena, Wael M. Rabeh

**Affiliations:** 1 Science Division, New York University Abu Dhabi, PO Box 129188, Abu Dhabi, United Arab Emirates; 2 G42 Healthcare Omics Excellence Center, Masdar City, Abu Dhabi, United Arabes Emirates; 3 UESB - Universidade Estatudal Do Sudoeste da Bahia. Deparmento de Ciencias Biologicas; 4 Horticultural Sciences Department, University of Florida, Gainesville, FL 32611, U.S.A.; 5 Kirby Institute, University of NSW, Sydney, NSW 2052. Australia; 6 Victoria University, Footscray Park Campus, Melbourne, VIC, 3134, Australia; 7 Aegros Therapeutics Pty Ltd, 5-6 Eden Park Drive, Macquarie Park, NSW 2113, Australia

**Keywords:** 3CLpro, Antivirals, Flavonoids, Luteolin, micronutrients, SARS-CoV-2

## Abstract

SARS-CoV-2 was first discovered in 2019 and has disseminated throughout the globe to pandemic levels, imposing significant health and economic burdens. Although vaccines against SARS-CoV-2 have been developed, their long-term efficacy and specificity have not been determined, and antiviral drugs remain necessary. Flavonoids, which are commonly found in plants, fruits, and vegetables and are part of the human diet, have attracted considerable attention as potential therapeutic agents due to their antiviral and antimicrobial activities and effects on other biological activities, such as inflammation. The present study uses a combination of biochemical, cellular, molecular dynamics, and molecular docking experiments to provide compelling evidence that the flavonoid luteolin (2-(3,4-dihydroxyphenyl)-5,7-dihydroxy-4H-chromen-4-one) has antiviral activity against SARS-CoV-2 3-chymotrypsin-like protease (3CLpro) that is synergistically enhanced by magnesium, zinc, and vitamin C. The IC_50_ of luteolin against 2 µM 3CLpro is 78 µM and decreases 10-fold to 7.6 µM in the presence of zinc, magnesium, and vitamin C. Thermodynamic stability analyses revealed that luteolin has minimal effects on the structure of 3CLpro, whereas metal ions and vitamin C significantly alter the thermodynamic stability of the protease. Interactome analysis uncovered potential host-virus interactions and functional clusters associated with luteolin activity, supporting the relevance of this flavone for combating SARS-CoV-2 infection. This comprehensive investigation sheds light on luteolin's therapeutic potential and provides insights into its mechanisms of action against SARS-CoV-2. The novel formulation of luteolin, magnesium, zinc, and vitamin C may be an effective avenue for treating COVID-19 patients.

## Introduction

Coronaviruses are a group of enveloped positive-sense RNA viruses that commonly infect human hosts during the influenza season [1]. They usually result in low-grade upper respiratory tract infections with symptoms similar to those of the common cold [2]. The development of therapies for coronavirus infections has largely been a neglected area of research due to their low seasonal prevalence. This status changed with the emergence of SARS-CoV-2 in Wuhan City, Hubei Province, Central China, in December 2019. The virus proliferated rapidly, and coronavirus disease 2019 (COVID-19) was announced as a global pandemic. SARS-CoV-2 has resulted in more deaths globally than its earlier counterparts, SARS-CoV-1 and Middle East Respiratory Syndrome (MERS), which emerged in 2002 and 2012, respectively, and did not develop to a pandemic scale. The dissemination and transmission of several variants of SARS-CoV-2 within the span of a few years caused unprecedented damage to the global economy and livelihoods.

Coronaviruses use the spike (S) protein on the surface of the viral capsid to enter host cells. The S protein binds angiotensin-converting enzyme II (ACE2) on type II alveolar cells, leading to the fusion of the viral and host cell membranes to deliver viral RNA into the host cytoplasm [3]. The viral RNA encodes the viral machinery and structural elements required for viral replication and enters the cell nucleus for replication [4]. ORF1ab, the largest viral gene, encodes overlapping open reading frames called PP1a and PP1ab. These polyproteins are cleaved into 16 nonstructural proteins (nsps1-16) by the main protease, which is also called 3-chymotrypsin-like protease (3CLpro), and papain-like protease (PLpro) [5,6]. Because processing by 3CLpro (nsp5) and PLpro (nsp3) is required for nsp maturation, these proteases play significant roles in viral transcription, replication, proteolytic processing, suppression of host immune responses, and host gene expression [7–9]. Therefore, they are attractive targets for therapeutic strategies for SARS-CoV-2.

The structural fold of 3CLpro is identical among betacoronaviruses, and the active site contains a conserved catalytic dyad (His41 and Cys145) that facilitates the proteolytic reaction. The monomer is split into three domains. Domains I (residues 10-96) and II (residues 102-180) form a five-stranded antiparallel β-barrel structure with a chymotrypsin-like scaffold [10,11]. Domain III (residues 200-303), located at the C-terminus, comprises a cluster of five α-helices connected to Domain II by a long loop (residues 181-199). In contrast with the traditional Ser-His-Asp triad of chymotrypsin, 3CLpro of SARS-CoV-2 has a catalytic Cys-His dyad. C145, which is located in domain II, engages in H-bonding interactions at 2.5 Å with the backbone carbonyl carbon of glutamine of the peptide substrate. H41 is part of domain I [12,13].

3CLpro is one of the most explored drug targets for SARS-CoV-2, as inhibiting it would prevent further viral proliferation. According to the World Health Organization (WHO), there is no specific therapy for SARS-CoV-2 that has undergone randomized clinical trials. Almost all antiviral inhibitors have been repurposed, including non-nucleoside inhibitors (Ribavirin, Favipiravir, Remdesivir, and Galidesivir) and protease and S protein inhibitors (Disulfiram, Lopinavir, Ritonavir, Nafamostat, and Griffithsin). While most of these inhibitors are Food and Drug Administration (FDA)-approved, few have provided clear-cut benefits in clinical trials or proved to be specific against SARS-CoV-2. Thus, designing selective drug candidates or identifying novel drugs against SARS-CoV-2 proteins through small-molecule screening and structure-based drug design could provide an important perspective for developing antivirals against COVID-19 [14,15].

Vitamins, including vitamin C, and micronutrients, including zinc and magnesium, have been shown to play essential roles in various bodily functions, including immune response [16–30]. It is known that vitamin C concentration declines during infections, where vitamin C supplementation was found to improve the immune system [17,21]. Likewise, zinc is an essential trace element for maintaining immune function, and zinc deficiency is common due to lifestyle, age, and disease-mediated factors [16,19,23–34]. Consequently, zinc could impair antiviral immunity. Magnesium is another micronutrient essential for many biochemical reactions in the body, including those that regulate immune function [18,33,34]. It supports overall immune health and can help reduce inflammation in response to viral infections. Therefore, maintaining physiological concentrations of micronutrients could play an important role in the function of the immune system and host resistance to infectious agents, where vitamin C and zinc have been shown to reduce the incidence and improve the outcome of viral infections.

In the present study, the structure of 3CLpro of SARS-CoV-2 was used to virtually screen small molecules that can bind and tightly interact with the protease’s active site residues [35–41]. The ability of the computationally identified small molecules to bind and inhibit SARS-CoV-2 3CLpro was then verified to support the further development of antiviral therapeutics. Among the molecules screened, the phytochemical luteolin (2-(3,4-dihydroxyphenyl)-5,7-dihydroxy-4H-chromen-4-one), also called digitoflavone or flacitran, inhibited SARS-CoV-2 3CLpro. Luteolin also exhibited antiviral activity against live virus (SARS-CoV-2) in a cell-based assay. In addition, the combined presence of magnesium, zinc, and vitamin C (L-ascorbate) synergistically enhanced the activity of luteolin. Luteolin is the latest in a number of phytochemicals that have shown good antiviral activity against the SARS-CoV-2 S or protease proteins. An antiviral formulation comprising luteolin in combination with metal ions and vitamin C may provide much-needed relief to those suffering from COVID-19 and potentially future related coronaviral infections.

## Results

### Screening of inhibitors against 3CLpro

**Figure 1:**
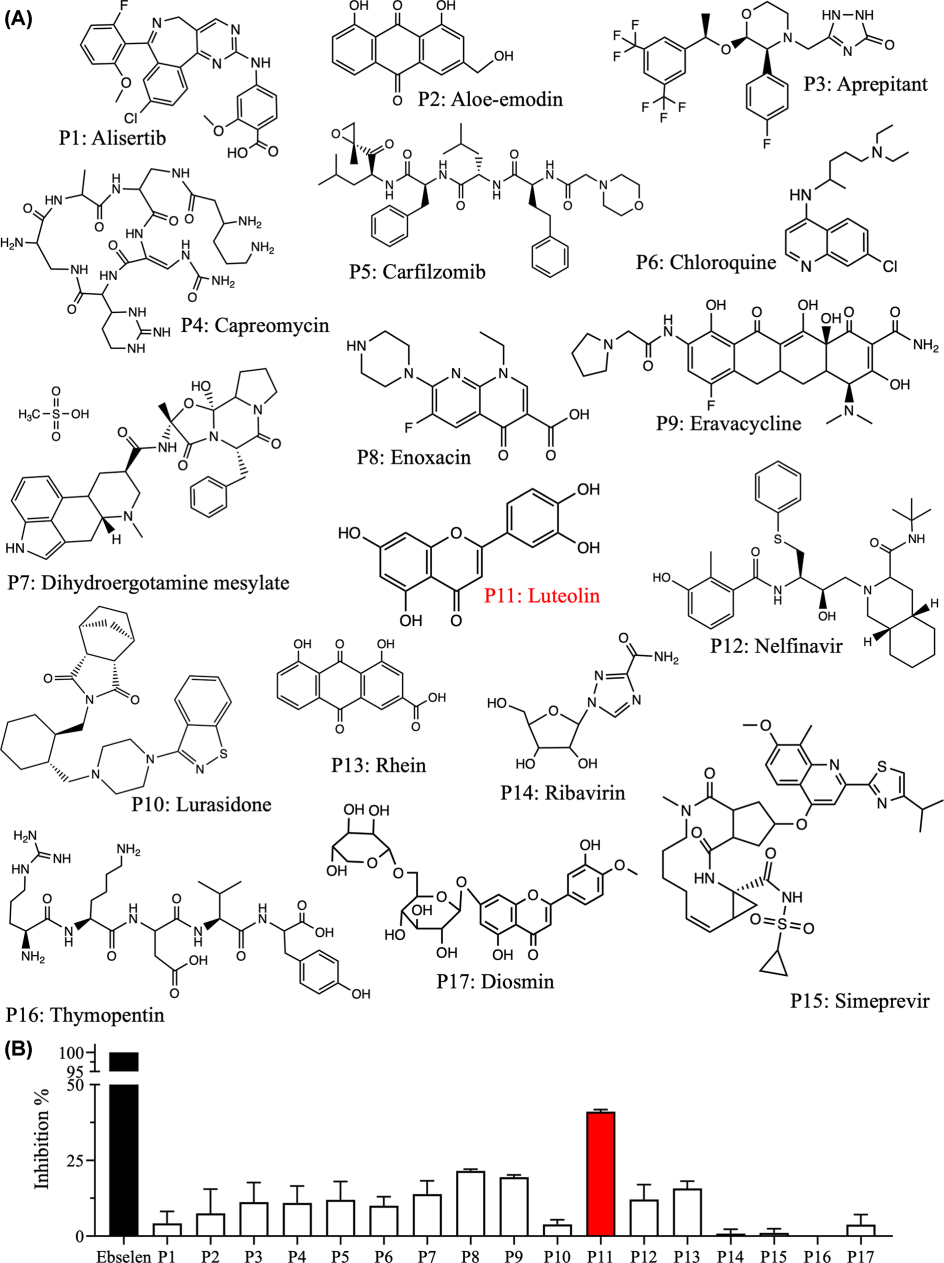
Screening of different potential inhibitors (P1-P17) of 3CLpro (**A**) Chemical structures of the small molecules selected from multiple *in silico* drug screening libraries [36–41]. (**B**) Bar plot of the percentage inhibition of SARS-CoV-2 3CLpro at a fixed inhibitor concentration of 25 µM. The percent inhibition was normalized to the activity of 3CLpro in the absence of inhibitors. Ebselen was used as a positive control that completely inhibits 3CLpro activity. Luteolin (P11, red bar) had the highest 3CLpro inhibition capacity. Data are the mean ± SD, *n*=3.

To screen potential inhibitors of SARS-COV2 3CLpro, a shortlist of small molecules was selected based on previous computational studies. These studies applied several computational modeling and bioinformatics approaches to diverse libraries of small molecules, including a library of 14,064 marine natural products [39], a collection of 43 flavonoids [40], FDA-approved drugs and antiviral compounds [38,41], and libraries of known bioactive compounds [36,37], to identify small molecules with high affinity to SARS-CoV-2 3CLpro. Ultimately, 17 small molecules were selected for biochemical testing of their abilities to inhibit SARS‐CoV-2 3CLpro (Figure 1A).

First, inhibition was assessed in the FRET protease assay using a fixed small-molecule concentration of 25 µM and a fixed peptide substrate concentration of 60 μM in a buffer containing 20% (v/v) DMSO at 30°C. We previously reported that 20% DMSO is the most suitable concentration for enhancing the solubility of the peptide substrate and small-molecule inhibitors in the 3CLpro assay [42]. The percentage inhibition was determined by comparing 3CLpro activity in the presence of inhibitor to the activity in the absence of the inhibitor (Figure 1B). To validate the ability of the protease assay to identify potential inhibitors of SARS-CoV2 3CLpro, the potent 3CLpro inhibitor ebselen was used as a positive control [43–47]. Among the 17 potential inhibitors (*P*), luteolin (*P*11) had the greatest inhibitory effect on 3CLpro, with an inhibition capacity of 41% at 25 µM (Figure 1B).

**Figure 2:**
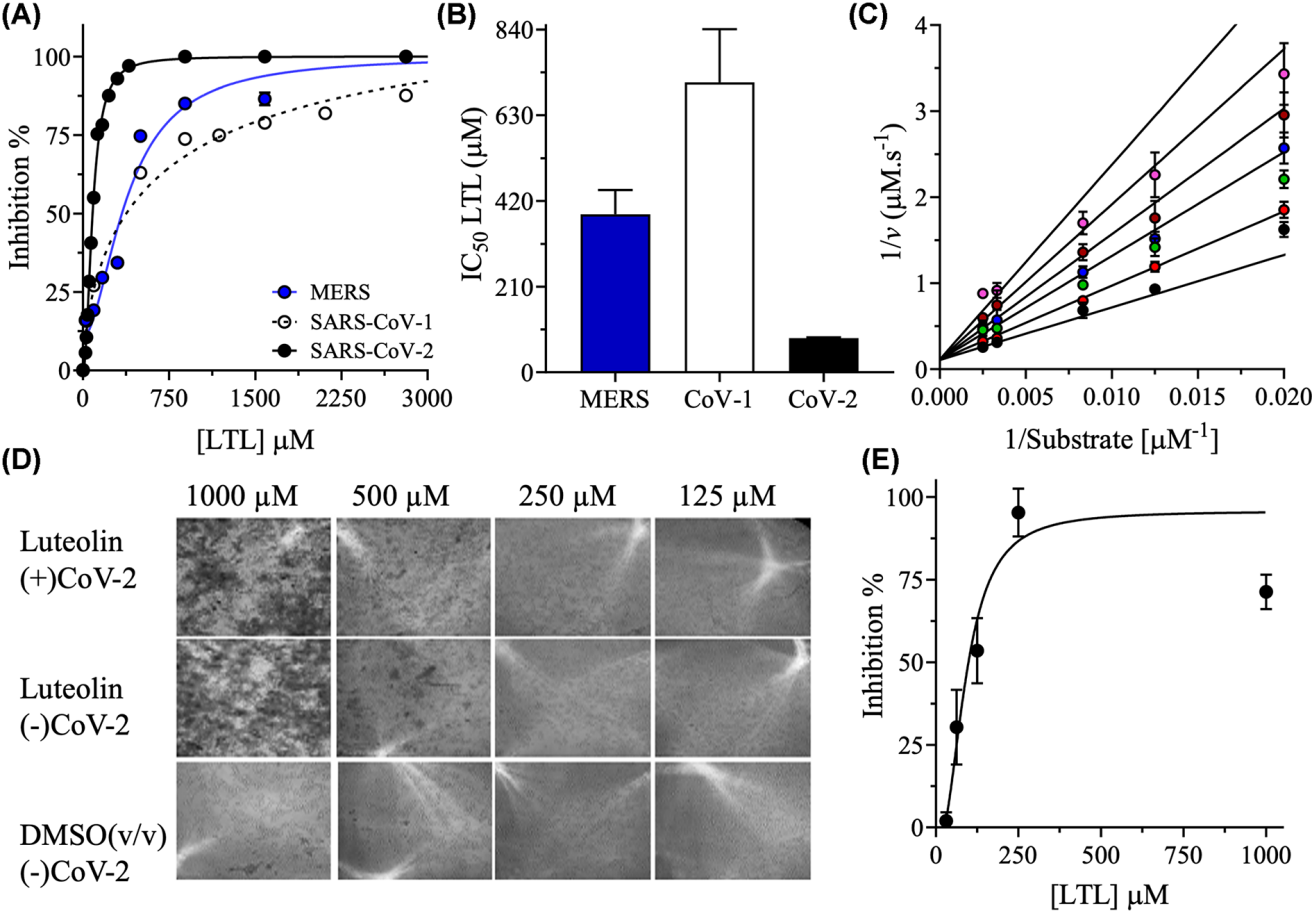
Inhibition of SARS-CoV-2 3CLpro by luteolin (**A**) Inhibition curves obtained by titrating luteolin against a fixed concentration of 2 µM 3CLpro from MERS (blue dots), SARS-CoV-1 (black circles), and SARS-CoV-2 (black dots). Assays were performed in 50 mM HEPES, pH 7.5, 1 mM EDTA, 1 mM TCEP, and 20% (v/v) DMSO at 30°C. The peptide substrate concentration was fixed at 60 μM. (**B**) Bar plot of the IC_50_ values of luteolin against 3CLpro from MERS (blue), SARS-CoV-1 (white), and SARS-CoV-2 (black) determined from the curves in (A). (**C**) Double-reciprocal plots of the competitive inhibition pattern obtained upon varying the concentration of the peptide substrate at 50, 80, 120, 300, and 400 µM at variable fixed concentrations of luteolin of 40, 95, 170, 225, 300, and 400 µM. Data are the mean ± SD, *n*=3. (**D**) The ability of luteolin to inhibit SARS-CoV-2 infection in permissive HEK293T cells stably expressing human ACE2 and TMPRSS2 (HekAT24) was assessed by high-content fluorescence microscopy. The bright-field images in the upper row show the effect of luteolin concentrations of 125, 250, 500, and 1000 µM on viral cytopathicity. The middle and bottom rows of images were obtained in the absence of SARS-CoV-2 and in the absence of virus and DMSO, respectively. Data are the mean ± SD, *n*=4. (**E**) Inhibition curve of luteolin titration against live SARS-CoV-2; the curve was used to calculate the IC_50_ of luteolin in the live virus assay.

Next, the half-maximal inhibitory concentrations (IC_50_s) of luteolin against 3CLpro enzymes of different coronaviruses – MERS, SARS-CoV-1, and SARS-CoV-2-were evaluated using a range of luteolin concentrations between 0.5 µM and 3 mM. The concentrations of 3CLpro and the peptide substrate were fixed at 2 µM and 60 µM, respectively (Figure 2A). Luteolin had IC_50_s of 388 ± 59 µM, 711 ± 130 µM, and 78 ± 2 µM against MERS 3CLpro, SARS-CoV-1 3CLpro, and SARS-CoV-2 3CLpro, respectively (Figure 2B).

Finally, the kinetics of inhibition of SARS-CoV-2 3CLpro were determined. A competitive inhibition pattern in which luteolin competed against the peptide substrate was observed when the concentration of luteolin was varied from 40 to 400 µM at different fixed concentrations of the peptide substrate ranging from 50 to 400 µM (Figure 2C). An inhibition constant (*K*_i_) of luteolin of 93 ± 12 µM was calculated when the data were fit to (eqn 1) for competitive inhibition. The apparent Michaelis constant (*K*_a_) for the peptide substrate in the presence of luteolin was 406 ± 48 µM, ∼6-fold higher than the Michaelis constant (*K*_m_) of 66 ± 7 µM for the peptide substrate in the absence of inhibitor [13,42]. The higher value of *K*_a_ is consistent with a pattern of competitive inhibition in which luteolin binds and competes with the peptide substrate for the active site of 3CLpro.

### Luteolin has SARS-CoV-2 antiviral activity in HEK293T cells

The antiviral activity of luteolin was assessed in a live SARS CoV-2 infection assay in ACE2-TMPRSS2-expressing HEK293T cells. The cells were incubated with different luteolin concentrations before infection with the ancestral strain of SARS-CoV-2 (A2.2). Infections in the presence of the carrier, DMSO, were performed in parallel to account for any carrier-induced cytotoxicity. Twenty hours after infection, the virus-induced cytopathic effect (CPE) was quantified by high-content microscopy (Figure 2D). Luteolin inhibited SARS-CoV-2 infection in a dose-dependent manner, with an IC_50_ value of 90 ± 11 µM in the live virus assay (Figure 2E). This IC_50_ value is similar to the IC_50_ value of 78 ± 2 µM obtained in the in vitro biochemical enzymatic assay and strongly supports the effectiveness of luteolin against SARS-CoV-2.

### Luteolin and micronutrients synergistically inhibit SARS-CoV-2 3CLpro

**Figure 3:**
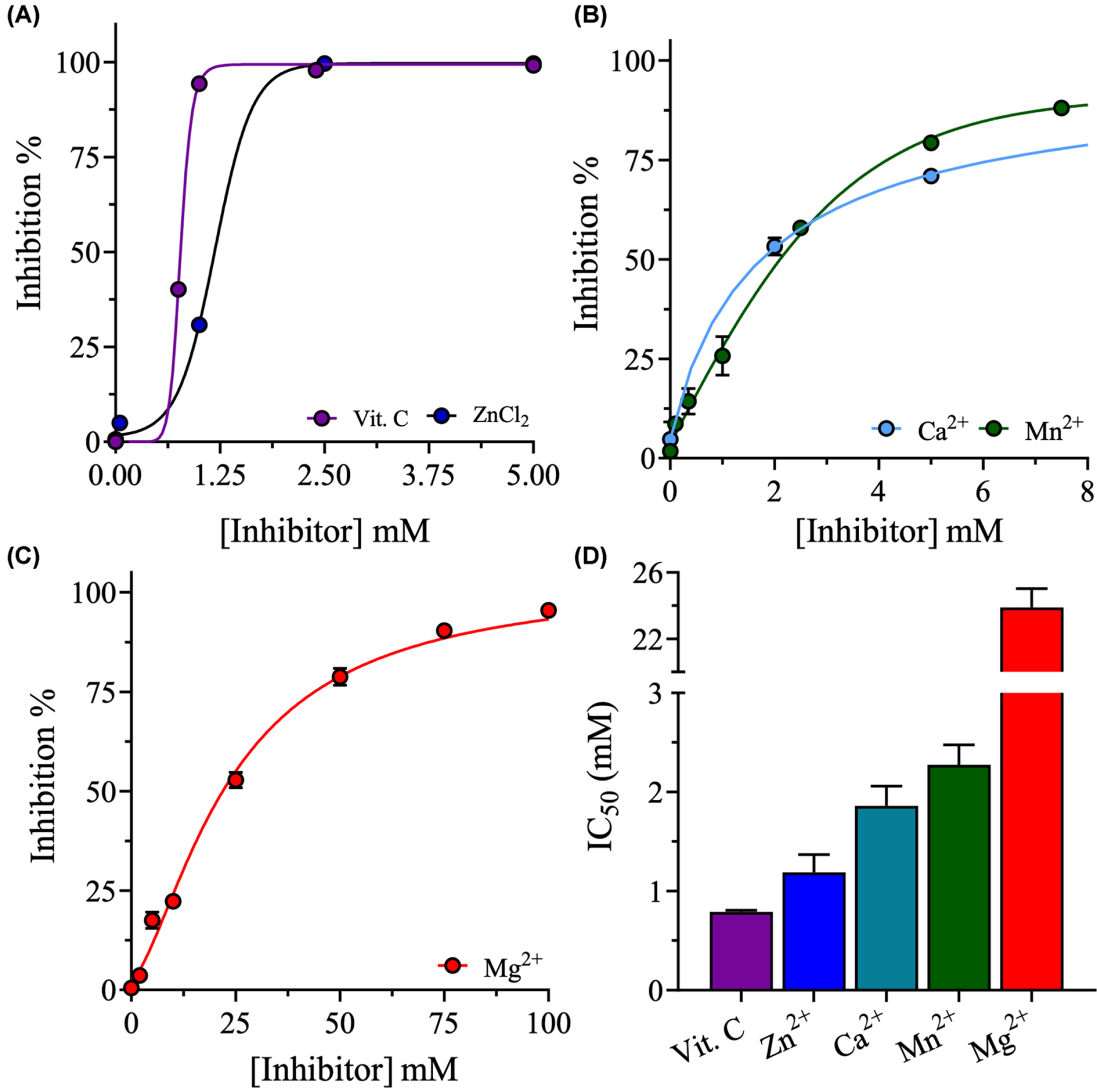
The inhibitory effects of vitamin C and metal ions on SARS-CoV-2 3CLpro activity (**A-C**) Inhibition curves of vitamin C and metal chlorides of Zn^2+^, Ca^2+^, Mn^2+^, and Mg^2+^ against 3CLpro. Enzymatic assays were performed in buffer containing 20% (v/v) DMSO at 30°C. The concentrations of 3CLpro and peptide substrate were fixed at 2 and 60 μM, respectively. The inhibition percentage was normalized to that of 3CLpro in the absence of micronutrients. (**D**) Bar plot of the IC_50_s of vitamin C and metal chlorides against SARS-CoV-2 3CLpro acquired from the inhibition curves in A–C. Data are the mean ± SD, *n*=3.

To identify molecules that may enhance the inhibitory effect of luteolin on SARS-CoV-2 3CLpro, the inhibitory effects of various metal ions and vitamins (micronutrients) on 3CLpro were evaluated. First, the IC_50_ values of vitamin C and metal chlorides of zinc, manganese, magnesium, and calcium were determined in the absence of luteolin. Vitamin C and ZnCl_2_ had the lowest IC_50_s against 3CLpro: 0.8 ± 0.02 mM and 1.1 ± 0.2 mM, respectively (Figure 3A). CaCl_2_ and MnCl_2_ had the second lowest IC_50_ values: 1.9 ± 0.2 mM and 2.3 ± 0.2 mM, respectively (Figure 3B). MgCl_2_ had the highest IC_50_, 24 ± 1.1 mM, and thus had the weakest inhibitory effects on 3CLpro activity (Figure 3C).

**Figure 4:**
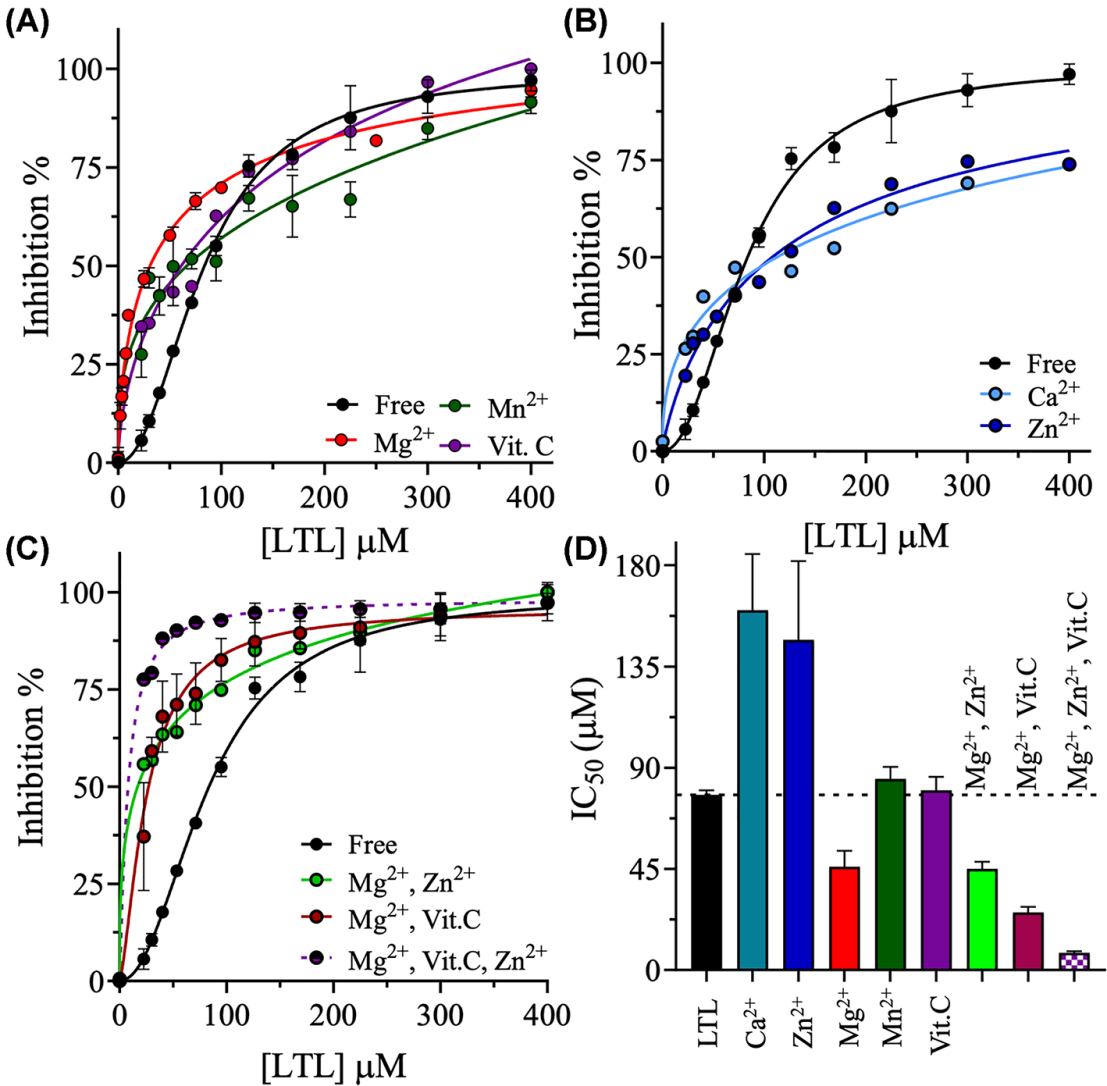
Synergistic inhibition of SARS-CoV-2 by luteolin and micronutrients (**A,B**) Inhibition curves of luteolin in the absence or presence of 3.5 mM Mg^2+^, 0.5 mM Ca^2+^, 0.3 mM Zn^2+^, 2.3 mM Mn^2+^, or 0.3 mM vitamin C. The luteolin titration curves were acquired as described in Figure 2A. (**C**) Inhibition curves of luteolin in the absence or presence of multiple micronutrients. The concentrations of the micronutrients were the same as those in panels A,B. (**D**) Bar graph of the IC_50_s of luteolin in the absence or presence of various metal ions and vitamin C, which were determined from the titration plots of luteolin in A–C. Data are the mean ± SD, *n*=3.

Because all of the evaluated micronutrients inhibited SARS-CoV-2 3CLpro under these conditions, their potential synergism with luteolin to inhibit 3CLpro was evaluated. The concentration of each micronutrient was fixed at its IC_50_ value in the enzymatic assay, and the IC_50_ value of luteolin was determined by varying the concentration of luteolin from 3 µM to 400 µM, eight times lower than the luteolin concentrations in the absence of micronutrients. The IC_50_ of luteolin decreased by 1.7-fold, from 78 ± 2 µM to 46 ± 7 µM, in the presence of magnesium (Figure 4A,D). The IC_50_ of luteolin did not change in the presence of manganese or vitamin C, with values of 85 ± 5 µM and 80 ± 6 µM, respectively (Figure 4A,D). However, in the presence of zinc or calcium, the IC_50_ of luteolin increased by 2-fold to 147 ± 35 µM and 160 ± 25 µM, respectively (Figure 4B,D).

To further explore conditions for improving the efficacy of luteolin against SARS-CoV-2 3CLpro, the effects of combinations of different micronutrients were assessed. The IC_50_ of luteolin was 45 ± 2 µM in the presence of both magnesium and zinc (Figure 4C,D), similar to the IC_50_ of luteolin in the presence of magnesium only, which suggested that zinc does not influence the synergy of luteolin and magnesium. Next, the synergy of magnesium was investigated in the presence of vitamin C, which further reduced the IC_50_ of luteolin to 26 ± 3 µM (Figure 4C,D). Finally, the IC_50_ of luteolin was investigated in the presence of magnesium, zinc, and vitamin C, which reduced the IC_50_ of luteolin against 3CLpro to 7.6 ± 0.8 µM. Thus, the presence of the three micronutrients decreased the IC_50_ of luteolin against SARS-CoV-2 3Cpro by 10-fold compared to its value in the absence of micronutrients, highlighting the powerful synergistic effect of this cocktail (Figure 4C,D).

### Thermodynamic stability of 3CLpro in the presence of luteolin and micronutrients

**Figure 5:**
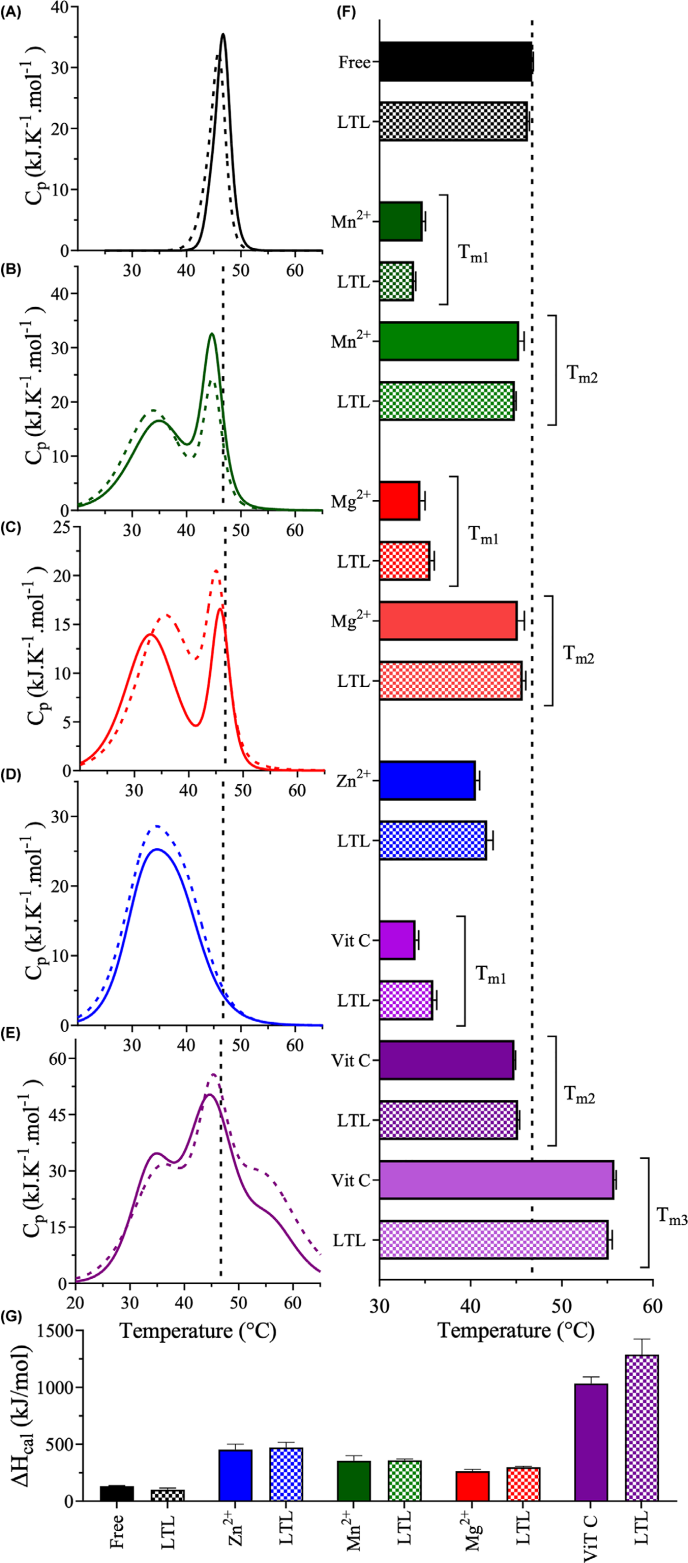
Thermodynamic stability of 3CLpro in the presence of inhibitors DSC thermograms of 3CLpro were acquired in the absence (solid line) or presence of 100 µM luteolin (dotted line) and (**A**) in the absence of micronutrients (black), (**B**) in the presence of 2.3 mM Mn^2+^ (green), (**C**) in the presence of 3.5 mM Mg^2+^ (red), (**D**) in the presence of 0.3 mM Zn^2+^ (blue), and (**E**) in the presence of 0.3 mM vitamin C (purple). The temperature was increased from 15°C to 75°C at a scanning rate of 1°C/min in 50 mM HEPES, pH 7.5, 1 mM EDTA, 1 mM TCEP, and 20% (v/v) DMSO. (**F**) Bar plot of the *T*_m_ values calculated at the apex of the melting peaks in the absence (solid bar) or presence of 100 µM luteolin (checkered bar) with or without micronutrients. (**G**) Bar plot of Δ*H*_cal_ calculated from the area under the thermographic peak in the absence (solid bar) or presence of 100 µM luteolin (checkered bar) with or without micronutrients. Data are the mean ± SD, *n*=3.

The thermodynamic stability of 3CLpro in the absence or presence of luteolin, metal ions, and vitamin C was assessed by differential scanning calorimetry (DSC) (Figure 5). The thermograms of 3CLpro in the absence or presence of 100 µM luteolin exhibited a single-state transition, and the melting point (*T*_m_), determined at the apex of the peak, was 46.7 ± 0.1°C in both cases (Figure 5A,F). The differences between the thermograms were minor; the peak in the presence of luteolin was narrower than the peak in the absence of luteolin (Figure 5A).

To investigate the effects of metal ions on the thermodynamic stability of 3CLpro, DSC scans of 3CLpro were obtained in the presence of metal ions at concentrations close to their physiological concentrations. Two-transition thermograms were observed for 3CLpro in the presence of 2.3 mM manganese or 3.5 mM magnesium (Figure 5B,C). An early transition that was not present in the apo-state appeared in the presence of manganese or magnesium, indicating an early unfolding event. The *T*_m_ of the first transition (*T*_m1_) was 35 ± 0.3°C in the presence of either manganese or magnesium, indicating reduced stability compared with apo-3CLpro (Figure 5F). The *T*_m_ of the second transition (*T*_m2_), 45 ± 0.5°C, was similar to that obtained from the single peak of apo-3CLpro. However, in the presence of 0.3 mM zinc, the thermogram of 3CLpro exhibited a single transition with a *T*_m_ of 41 ± 0.4°C, approximately 6°C lower than that of apo-3CLpro (Figure 5D,F). Finally, the thermodynamic stability of 3CLpro was assessed in the presence of both luteolin and metal ions. The addition of luteolin had a minimal effect on the thermograms of 3CLpro in the presence of metal ions, with *T*_m_ values similar to those in the presence of metal ions without luteolin (Figure 5F).

Vitamin C had distinct effects on the thermodynamic stability of 3CLpro compared to the metal ions. The thermogram of 3CLpro in the presence of 0.3 mM vitamin C exhibited a three-state transition with additional low and high transitions compared to apo-3CLpro (Figure 5E). The middle transition had a *T*_m2_ of 45 ± 0.7°C, similar to that of the single peak of apo-3CLpro (Figure 5F). The early transition had a *T*_m1_ of 34°C ± 0.3, similar to that of the early peaks observed in the presence of manganese or magnesium. The late transition, which was not observed in any of the other DSC scans, had a *T*_m3_ of 56 ± 0.2°C. Again, the addition of luteolin did not change the stability or shape of the thermographic peaks of 3CLpro in the presence of vitamin C.

Finally, the calorimetric enthalpy (Δ*H*_cal_) of unfolding was determined by calculating the area under the thermographic peak. Similar to the effects on *T*_m_ values, the presence of 100 µM luteolin had only a small effect on Δ*H*_cal_, which decreased from 133 ± 4 kJ/mol for the free enzyme 3CLpro to 101 ± 15 kJ/mol in the presence of luteolin (Figure 5G). However, Δ*H*_cal_ increased when metal ions were added in the absence of luteolin. The addition of magnesium, manganese, or zinc increased Δ*H*_cal_ by 2-, 3-, or 4-fold to 264 ± 15 kJ/mol, 356 ± 45 kJ/mol, or 453 ± 50 kJ/mol, respectively, and adding luteolin in the presence of metal ions did not have further significant effects on Δ*H*_cal_.

Consistent with the wide, three-transition thermogram of 3CLpro in the presence of vitamin C, the Δ*H*_cal_ of 3CLpro was most elevated in the presence of vitamin C (Figure 5E): 1034 ± 58 kJ/mol in the absence of luteolin (an 8-fold increase) or 1288 ± 136.3 kJ/mol in the presence of luteolin (a 10-fold increase) (Figure 5G). These results indicate that the stability of 3CLpro was not altered by the presence of luteolin but decreased greatly in the presence of metal ions and vitamin C.

### Docking and molecular dynamics simulations of SARS-CoV-2 3CLpro and luteolin

**Figure 6:**
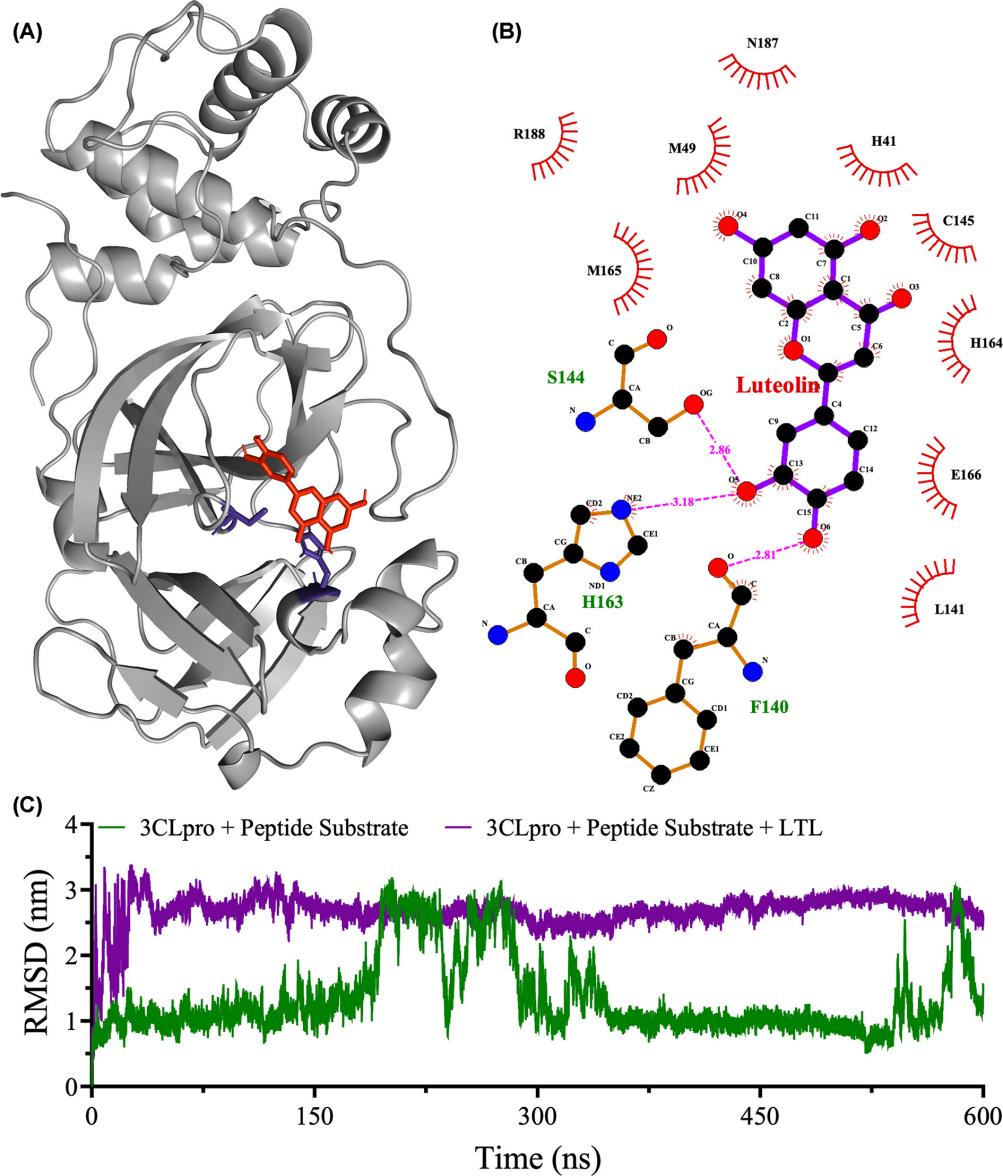
Molecular docking interactions of luteolin with SARS-CoV-2 3CLpro (**A**) Cartoon representation of 3CLpro with luteolin (red ball-and-stick structure). Luteolin binds in the 3CLpro active site; the catalytic dyad, His 41 and Cys145, is shown in purple ball-and-stick form. (**B**) 2D interaction map of luteolin with 3CLpro active site residues. Magenta dashed lines indicate hydrogen bonds, and semicircles show hydrophobic and electrostatic interactions of luteolin with 3CLpro. (**C**) RMSD variations of the peptide substrate in 600-ns molecular dynamics simulations of 3CLpro in complex with the peptide substrate in the absence (green line) or presence of luteolin (purple line).

Docking experiments were carried out to generate a complex between SARS-CoV-2 3CLpro (PDB code 7E19) and luteolin (Figure 6A) [48]. The docking experiments were guided by the competitive inhibition of 3CLpro by luteolin (Figure 2C), which suggested that luteolin binds in the catalytic pocket. Examining the binding affinity between 3CLpro and luteolin is crucial for understanding the molecular recognition of the 3CLpro-luteolin complex [49]. The computational analysis revealed a protein-luteolin affinity energy of −7.7 kcal/mol within the catalytic pocket of 3CLpro. The 2D interaction map indicated that three hydrogen bonds are formed between luteolin and F140, S144, and H163 of 3CLpro (Figure 6B): between O6 of luteolin and the backbone carbonyl oxygen of F140 at 2.81 Å; between O5 of luteolin and the side chain hydroxyl of S144 at 2.86 Å; and between O5 of luteolin and the NE2 atom of the imidazole ring of H163 at 3.18 Å. Furthermore, luteolin exhibits hydrophobic interactions with residues M49, L141, and M165 and polar interactions with residues H41, C145, H164, E166, N187, and R188 (Figure 6B).

To validate the docking methodology, a re-docking experiment was conducted using the crystallographic ligand (inhibitor) HUO from the same 3CLpro structure utilized in all docking experiments (PDB code 7E19) [48]. The root-mean-square deviation (RMSD) between the positions of the docked ligand and the crystallized ligand was 1.54 Å, below the cut-off of 2.0 Å recommended in prior studies [50,51].

A 600-ns molecular dynamics (MD) simulation was conducted on the 3CLpro-substrate complex in the absence and presence of luteolin. Luteolin was included in the proximity of the peptide substrate within the catalytic pocket of 3CLpro. For the 3CLpro-peptide substrate complex, the RMSD graph indicated that the peptide substrate inside the catalytic pocket of 3CLpro was stable from the initiation to the termination of the MD simulation (Figure 6C). The RMSD value of the peptide substrate was much higher in the 3CLpro-substrate-luteolin complex, indicating significant destabilization of the binding of the peptide substrate in the presence of luteolin during the MD simulation (Figure 6C). The 3CLpro-substrate-luteolin complex underwent a huge conformational change at the beginning of the simulation, which increased the peptide substrate RMSD from 0.6 to ∼3 nm. The 3CLpro-substrate complex also exhibited an increase the substrate RMSD at the beginning of the simulation, from 0.6 to 1.3 nm; thereafter, the RMSD of the peptide substrate remained relatively constant throughout the simulation time, with the exception of high dynamics between 190 and 290 ns. The MD video in the supplementary material shows that luteolin (blue) consistently occupied the catalytic pocket of 3CLpro (red) throughout the MD simulation, whereas the peptide substrate (green) was released from the catalytic pocket. These observations strongly support the competitive inhibition mechanism of luteolin determined in the kinetics experiments.

## Interactome of 3CLpro with human proteins, luteolin, vitamin C, magnesium, and zinc

**Figure 7:**
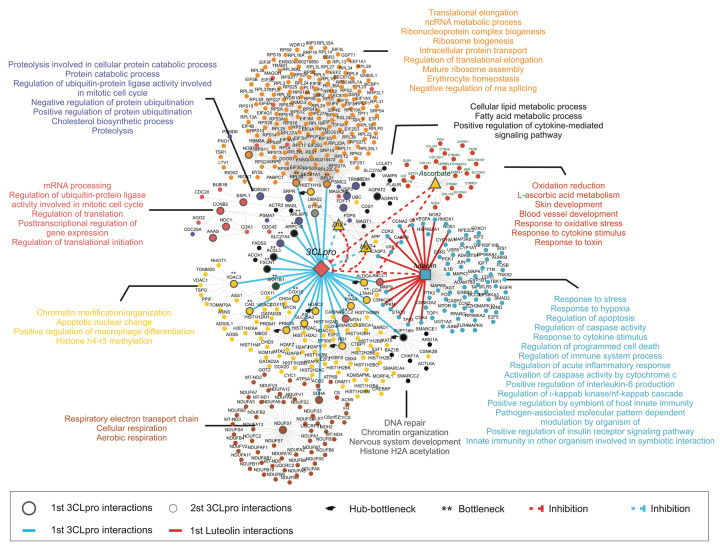
Interactome of 3CLpro and luteolin with human core proteins The large and small circles correspond to first- and second-degree interactions with human core proteins, respectively. These interactions are also highlighted with solid red and cyan connectors, respectively. The hub-bottlenecks and bottleneck are indicated by black pointing hands and double asterisks (**), respectively. The red dotted connectors represent the inhibitory potential of luteolin and other inhibitors against 3CLpro.

The virus–host interaction during infection is the product of complex protein-protein interactions that may be influenced by non-protein molecules such as metal ions, luteolin, and vitamin C. The analysis of the interactions of SARS-CoV-2 3CLpro, metal ions, luteolin, and vitamin C with human proteins revealed an interactome comprising 1978 nodes, 33,169 connectors, 479 hub (H), 127 bottleneck (B), and 157 hub-bottleneck (HB) protein candidates (complete data not shown). Restricting the interactome to first- and second-degree interactions (only for central proteins: H, B, or HB) of luteolin, metal ions, vitamin C, and 3CLpro reduced it to 448 nodes, 766 connectors, 200 H, 58 B, and 84 HB proteins (Figure 7). The first-degree interactions of 3CLpro (i.e., interact directly with 3CLpro) include 23 proteins: 7 HB proteins (ALDOA, HDAC2, PRMT5, RB1, RPL21, RPL30, and SUPT16H) and 16 B proteins (ABCC1, ABCC2, ACSL3, AKR1B1, ARL6IP5, ARPC1B, CAD, COX15, FSCN1, LTA4H, PIAS4, SDHA, SLC25A3, SLC27A4, STT3A, and VDAC3) (Figure 7). In Figure 7, the HB proteins are indicated by a black hand, and the B proteins are indicated by double asterisks (**). Among these proteins, SUPT16H, ALDOA, PIAS4, RB1, FSCN1, PRMT5, and LTA4H have second-degree interactions with luteolin through the proteins CDK2, CDK4, CSNK2A1, CSNK2A2, MMP9, PKM, STAT3, TOP1, TP53, and VEGFA (Figure 7). Moreover, the ATP-binding cassette, subfamily C protein ABCC1 has first-degree interactions with both 3CLpro and luteolin, indicating that ABCC1 is important for viral infection. With respect to the possible role of metal ions in viral infection, Mg^2+^ and Zn^2+^ ions each interacted with 16 and 14 proteins and 19 and 16 connectors, respectively. These findings demonstrate the importance of metal ions in different cellular processes during host-virus interaction.

## Cluster analysis of protein–protein interactions

Nine functional clusters (clusters 1–9) enriched in biological processes were identified (Figure 7). These biological processes were mainly associated with mature ribosome assembly (Cluster 1), cellular lipid metabolism (Cluster 2), response to oxidative stress (Cluster 3), regulation of cell death (Cluster 4), chromatin organization (Clusters 5 and 7), cellular respiration (Cluster 6), mRNA processing (Cluster 8), and negative and positive regulation of protein ubiquitination (Cluster 9). Interestingly, luteolin is directly or indirectly connected to some of these processes through interactome networks, underpinning its potential relevance for treating SARS-CoV-2 infections. Several of the host proteins in cluster 4 that interact with luteolin are involved in processes that are highly relevant to SARS-CoV-2 infection: regulation of apoptosis, response to stress, regulation of programmed cell death, response to hypoxia and cytokine stimulus, regulation of immune system process, anti-apoptosis, positive regulation of insulin receptor signaling pathway, regulation of caspase activity, regulation of acute inflammatory response, and positive regulation by symbiont of host innate immunity, among others (Figure 7). In Cluster 7, the proteins that directly interact with VDAC3, PRMT5, and RB1 are enriched in biological processes of chromatin modification/organization, apoptotic nuclear change, positive regulation of macrophage differentiation, and histone H4-R3 methylation. Moreover, the proteins that directly interact with NDUFS1_SDHA in Cluster 6 are involved in the respiratory electron transport chain, cellular respiration, and aerobic respiration, all of which have roles in host–virus interactions.

Cluster analysis and subsequent biological process enrichment analysis also showed that 3CLpro, the metal ions, vitamin C, and luteolin interact with a group of proteins with high connectivity that are mainly involved in processes related to chromatin assembly/disassembly, regulation of cell death, positive regulation of gene expression, epigenetic lung alveolus development, immune response, blood vessel development, regulation of apoptosis, and response to an organic substance. SARS-CoV-2 modulates these host processes to achieve infection (Figure 7).

## Discussion

*In silico* studies have proposed a variety of potential antiviral drugs for COVID-19 treatment. Given the rapid evolution of COVID-19 variants, it is critical to develop effective oral antiviral drugs that can successfully combat SARS-CoV-2 in the early stages of infection. Such antivirals could help reduce the severity of COVID-19 infections. This study evaluated the effects of different combinations of luteolin, metal ions, and vitamin C, i.e., molecules consumed as dietary supplements, against SARS-CoV-2. Luteolin is a flavonoid, an important class of phytochemicals that are commonly found in fruits and vegetables. Flavonoids possess antiviral, antimicrobial, anti-inflammatory, and anticancer activities [52]. Other flavonoids with antiviral properties include catechins, quercetin, baicalein, and kaempferol [53–57]. Luteolin has immunomodulatory, anti-inflammatory, and antiviral effects, making it an interesting candidate antiviral for SARS-CoV-2.

2-Phenyl-1-benzopyran-4-one is the basic skeleton of the flavonoid family, which includes a large class of molecules known as flavones. In addition to luteolin, natural flavones include 6-hydroxyflavones, wogonin, tangeritin, baicalein, apigenin, scutellarein, and chrysin [52,56,58,59]. The antiviral potential of flavones has been known since the 1990s, when Mucsi, Gyulai, and Béládi demonstrated that simultaneous treatment with apigenin and acyclovir enhances the antiviral effect of acyclovir against herpes simplex viruses 1 and 2 (HSV-1 and HSV-2) in cell culture [60]. Luteolin has antiviral effects on HIV-1 reactivation by inhibiting Clade B and C Tat-driven long terminal repeat (LTR) transactivation [61]. After the HIV-1 genome is integrated into the host genome, luteolin abrogates viral activity by interfering with the binding of pTEF-b to LTRs [61]. Luteolin can also prevent NF-κB activation, inhibit host factors involved in transcription, or inhibit viral mRNA translation [62].

In addition, luteolin significantly inhibits Epstein-Barr virus (EBV) reactivation in cells [63]. Luteolin suppresses the activities of the immediate-early genes Zta and Rta by releasing the bound transcription factor Sp1. In a study of the inhibitory effects of 400 natural products on EV71 and coxsackievirus A16 infections found that luteolin is a potent inhibitor of viral RNA replication [63,64]. Finally, luteolin or a luteolin-rich fraction has been shown previously to possess antiviral efficacy against SARS-CoV-1, Rhesus rotavirus, Chikungunya virus (CHIKV), and Japanese encephalitis virus (JEV) [65–68].

### Metal ions and vitamin C enhance the inhibitory effects of luteolin on 3CLpro

In the biochemical assay, luteolin inhibited the proteolytic activity of the 3CLpro proteases of MERS, SARS-CoV-1, and SARS-CoV-2. Although the 3CLpro of SARS-CoV-2 shares 96% and 87% protein sequence identity with those of SARS-CoV-1 and MERS, respectively [69], the IC_50_ of luteolin was lowest for the 3CLpro of SARS-CoV-2. Luteolin functioned as a competitive inhibitor of SARS-CoV-2 3CLpro with a *K*_i_ value of 90 µM, making it a potential antiviral agent (Figure 2A,C). To improve the inhibitory effect of luteolin, different metal chlorides and vitamin C were screened for synergistic effects on SARS-CoV-2 3CLpro. Magnesium, the most abundant metal ion in cells, was the weakest inhibitor among the metal ions assessed and decreased the IC_50_ of luteolin by 2-fold. Zinc, the second most abundant element in cells, was the most potent inhibitor of SARS-CoV-2 3CLpro among the metal ions tested. However, zinc increased the IC_50_ of luteolin, both in the presence and absence of vitamin C. Interestingly, the combination of zinc and magnesium decreased the IC_50_ of luteolin by 2-fold. Vitamin C is known for its physiological role in suppressing both the severity and speed of bacterial and viral infections [17]. Vitamin C did not reduce the IC_50_ of luteolin; however, the combination of vitamin C and magnesium decreased the IC_50_ of luteolin by 3-fold. The greatest synergistic effects were observed when luteolin was combined with zinc, magnesium, and vitamin C, which reduced the IC_50_ of luteolin against SARS-CoV-2 3CLpro by 10-fold.

### Mechanism of binding of luteolin to 3CLpro

The antiviral activity of luteolin against SARS-CoV-2 is supported by its ability to inhibit both the proteolytic activity of 3CLpro and the infection of HEK293T cells by live virus in our *in vivo* assay. The enzyme inhibition studies guided *in silico* docking experiments to determine the mode of binding of luteolin in the active site of SARS-CoV-2 3CLpro. The enzyme and cell assays demonstrated that luteolin is a competitive inhibitor; i.e., it competes with the peptide substrate for binding to the active site of 3CLpro. The *in silico* docking experiments showed direct interactions of luteolin with residues in the catalytic pocket of 3CLpro and an affinity energy of –7.7 kcal/mol, very similar to the energy of -8.0 kcal/mol when the crystallographic ligand HUO was re-docked [48]. The similarity of the affinity energies validates the *in silico* prediction of the affinity energy of luteolin. Luteolin interacts with H41 and C145, which form the conserved catalytic dyad [13,70], and H163, which plays a pivotal role in sustaining the catalytic activity of 3CLpro [12,71]. The RMSD plots obtained from the MD simulations further support the direct binding of luteolin to the catalytic pocket of 3CLpro and displacement of the peptide substrate, consistent with a competitive inhibition mechanism (Supplementary videos).

### Mechanistic insights into SARS-CoV-2 pathogenesis and the luteolin antiviral effects

It is important to delineate not only the molecular interactions of SARS-CoV-2 3CLpro with host proteins that may be relevant for drug discovery but also functional associations that impact SARS-CoV-2-induced pathogenesis. Luteolin inhibited the proteolytic activity of 3CLpro, which is crucial for SARS-CoV-2 replication, *in vitro* and *in vivo*. Moreover, luteolin is functionally associated with human host proteins that interact with SARS-CoV-2 3CLpro and are critical to host-virus interactions. The analysis of the triangular interactome network of human proteins, 3CLpro, and luteolin identified both functional and structural associations (Figure 7), providing insights into candidate proteins that could be druggable targets in future antiviral strategies. Seven high-confidence hub-bottleneck human proteins that interact directly with 3CLpro were identified. These hub-bottleneck proteins include the epigenetic regulator histone deacetylase 2 (HDAC2). A direct interaction of 3CLpro and HDAC2 is supported by a study in which affinity purification and mass spectrometry showed that HDAC2 interacts with NSP5/3CLpro [72]. Two other hub-bottleneck proteins, retinoblastoma protein (RB1) and protein arginine methyltransferase 5 (PRMT5), are histone proteins that are involved in the epigenetic regulation of SARS-CoV-2-mediated disease (Figure 7). It has been suggested that 3CLpro epigenetically regulates the human gene machinery by inhibiting HDAC2 transport into the nucleus, potentially impacting the role of HDAC2 in mediating inflammation and the interferon (IFN) response [73,74]. SARS-CoV-2 3CLpro may also actively block IFN induction to blunt the host antiviral immune response [73,74]. PRMT5 regulates histone methylation and the methylation of key regulatory proteins involved in RNA splicing, cell cycle, and cell death [75]. Modification of viral proteins by host PRMTs supports the viral life cycle. PRMT1 methylates the SARS-CoV-2 nucleocapsid (N) protein, which reduces the RNA-binding activity of the N protein and suppresses stress granule (SG) formation [76]. The interactome data indicate that inhibiting PRMT1 reduces N-methylation, which is essential for viral production. Additionally, the interaction of NSP5/3CLpro with RB1 affects the cell cycle and SARS-CoV-1 cytotoxicity [77,78]. These findings underscore that SARS-CoV-2 manipulates the host’s epigenetic machinery to suppress IFN signaling genes, a common strategy among viruses.

The interactome analysis also revealed that SARS-CoV-2, 3CLpro, and luteolin interact within two prominent clusters exhibiting high connectivity and encompassing critical cellular processes (Figure 7, Clusters 4 and 7), including chromatin assembly/disassembly, regulation of cell cycle, programmed cell death, gene expression regulation, epigenetic control, lung alveolus development, immune response, blood vessel development, apoptosis regulation, and response to organic substances. For example, SARS-CoV-2 infection induces the phosphorylation of cyclin-dependent kinase-2 (CDK2), which is present in Cluster 4, reducing the activity of CDK2 and leading to cell-cycle arrest [79,80]. Although the efficacy of CDK inhibitors against COVID-19 remains uncertain, CDK inhibitors such as CGP-604747 may play a valuable role, particularly in lowering the expression of indicator genes for SARS-CoV-2-induced injury to lungs [79]. Some of the 9 clusters enriched in biological processes include not only essential bottleneck proteins but also hub-bottleneck proteins.

The interactome analysis of luteolin with human proteins and 3CLpro shed light on how the virus manipulates key human proteins and the interactions of luteolin with these proteins. Some of these viral interactions include proteins involved in lung alveolus development, suggesting roles of these interactions in pneumonia or acute respiratory distress syndrome (ARDS) that often accompany severe COVID-19 [5]. Luteolin may positively modulate genes that are critical for mitigating inflammation in lung injury. Additionally, the luteolin interactome network revealed direct interactions of luteolin with CDK-2 and -4, reaffirming their functional significance.

Moreover, luteolin inhibits the kinase CK2α′, which plays vital roles in cell proliferation, growth, and survival and in various cellular processes, including angiogenesis [81]. CK2 is implicated in numerous pathologies, including neurodegenerative diseases, inflammation, viral infections, parasite infections, and cancer. Luteolin’s ability to inhibit CK2α′ may position CK2α′ as a druggable target with potential therapeutic relevance to COVID-19. In addition, luteolin inhibits the matrix metalloproteinases (MMPs), MMP2 and MMP9, which are involved in SARS-CoV-2 pathogenesis [82]. MMP9 occupies a prominent position in the interactome network in Figure 7, levels of MMP9 and MMP2 in plasma are associated with COVID-19 mortality [83]. The synergy of luteolin with zinc and magnesium and the associations of these metal ions with NOS1 and NOS2 may help regulate cytokines involved in the cytokine storm seen in SARS-CoV-2 infections. Thus, combining luteolin with metal ions and vitamin C may be a valuable approach to prevent and treat SARS-CoV-2 infections by boosting immunity.

In conclusion, this analysis demonstrates that luteolin is a potential antiviral agent against SARS-CoV-2 and interacts synergistically with metal ions. The findings shed light on critical viral protein-protein interactions between the virus and the host and provide insights into the design of drugs based on luteolin that target viral protease activity [39]. While luteolin has an excellent safety profile, clinical trials are necessary to establish its full clinical efficacy, especially when combined with metal ions and vitamin C.

## Conclusion

We have proposed multiple micronutrients here for use in combination with luteolin, including zinc, magnesium, and vitamin C, due to their synergistic effects against SARS-CoV-2. Zinc acts as an antioxidant and enhances the immune response, aiding in the resistance to viral infections. Magnesium, often deficient due to lifestyle and modern agricultural practices, also plays a critical role in immune function. Additionally, vitamin C is known for its broad-spectrum antiviral activity. The recommended doses for luteolin and these micronutrients should follow the guidelines from the Dietary Supplement Fact Sheets of the Office of Dietary Supplements (ODS) at the National Institutes of Health (NIH). The proposed daily doses are 90.0 mg for adult men and 75.0 mg for adult women for vitamin C, 400 mg for adult men and 320 mg for adult women for magnesium, and 11.0 mg for adult men and 8.0 mg for adult women for zinc. However, Rigorous clinical trials, including randomized controlled trials and long-term safety studies, are necessary to evaluate the efficacy and safety of the antiviral supplements proposed here (luteolin, zinc, magnesium, and vitamin C) before they can be recommended for widespread use.

## Materials and methods

### Screening of inhibitors and IC_50_ calculation

The expression and purification of recombinant 3CLpro were described previously [84]. The proteolytic activity of 3CLpro was measured by using a fluorescence resonance energy transfer (FRET) assay. The assay monitored the fluorescence of the EDANS group, which is quenched by the acceptor DABCYL group in the peptide substrate DABCYL-KTSAVLQ↓SGFRKM-E(EDANS)-NH2 (the hydrolysis site is indicated by ↓) until liberated by hydrolysis [85,86]. The fluorogenic peptide was synthesized by GenScript (Piscataway, NJ, U.S.A.). The fluorescence of EDANS was monitored using excitation and emission wavelengths of 360 and 500 nm, respectively. Readings were obtained in a Cytation-5 multi-mode microplate reader (Biotek Instruments, Winooski, VT, U.S.A.). The reaction was performed in a 96-well plate in 20 mM HEPES pH 7.0 buffer, 150 mM NaCl, 1 mM EDTA, 1 mM TCEP, and 20% (v/v) dimethyl sulfoxide (DMSO) [42]. The concentrations of 3CLpro and the peptide substrate were fixed at 2 μM and 60 µM and, respectively. The reaction was monitored at 30°C for 5 min, and the rate was calculated from the increase in the fluorescence signal. The half-maximal inhibitory concentration (IC_50_) was determined by varying the concentrations of small-molecule inhibitors, metal ions, and vitamin C at fixed concentrations of the peptide substrate and 3CLpro. The cleavage rate was obtained by fitting the initial data to a linear equation using the Excel add-on package XLfit (IDBS Limited, Bridgewater, NJ, U.S.A.). The IC_50_ values were determined from the inhibition curves by non-linear regression analysis using GraphPad Prism software (version 9.1.2, GraphPad Software, U.S.A.). Reactions were analyzed in triplicate for each data point, and the values are displayed as the mean ± standard deviation (SD), *n*=3.

### Inhibition kinetics of luteolin

The pattern of inhibition of 3CLpro by luteolin was determined by monitoring 3CLpro activity in the presence of different concentrations of luteolin (40–400 µM) at different fixed concentrations of the peptide substrate (50–400 µM). The 3CLpro concentration was maintained at 2 µM. The proteolytic rate data were fit using the following competitive inhibition (eqn 1):

(1) $$v=\frac{V_{max}A}{K_{a}(1+(I/K_{i}))+A}$$

Where *v* is the initial velocity, *V*_max_ is the maximum velocity, *K_a_* is the apparent Michaelis constant, *A* is the substrate concentration, *I* is the inhibitor concentration, and *K_i_* is the inhibition constant. Data were fit using the global fitting analysis in the kinetics module of SigmaPlot (Systat Software, Inc. San Jose, California, U.S.A., www.sigmaplot.com). Triplicate reactions were analyzed for each data point, and the values are displayed as the mean ± standard deviation (SD), *n*=3.

### Cellular assay of SARS-CoV-2 inhibition

A high-content fluorescence microscopy approach was used to assess the ability of luteolin to inhibit SARS-CoV-2 infection in permissive cells. HEK293T cells stably expressing human ACE2 and TMPRSS2 (HekAT24) were generated as previously described [87]. Luteolin was diluted in cell culture medium to prepare 4× working stock solutions and then serially diluted further in the same medium to achieve a 2-fold dilution series. HekAT24 cells were trypsinized, stained with NucBlue in suspension (5% v/v), and seeded at 16,000 cells per well in a 384-well plate (Corning #CLS3985). Dilutions of luteolin were added to the cells, which were then incubated for 30 min at 37°C before adding an equal volume of virus solution. The cells were then incubated at 37°C for 24 h. Each test condition was run in quadruplicate. The cells were imaged using an InCell 2500 high-throughput microscope (Cytiva) with a 10× 0.45 NA CFI Plan Apo Lambda air objective. Acquired nuclei were counted using InCarta high-content image analysis software (Cytiva) to give a quantitative measure of the cytopathic effect. Virus inhibition/neutralization was calculated according to (eqn 2):

(2) $$\%N=D-\frac{(1-Q)\times 100}{D}$$

Where *Q* is the number of nuclei in the test well divided by the average number of nuclei in untreated uninfected controls and *D* = 1 − *Q* for wells containing cells infected with the virus but not treated with luteolin. Thus, the average nuclear counts for the infected and uninfected cell controls are defined as 0% and 100% inhibition, respectively. Wells containing cells treated with a given compound but no virus were included to account for cell death due to drug toxicity. The % inhibition by each compound concentration in infected wells was normalized to the % inhibition in wells with an equivalent compound concentration but no virus to yield the final inhibition values for each condition. The inhibition curves and the IC_50_ values were determined by non-linear regression analysis using GraphPad Prism software (version 9.1.2, GraphPad Software, U.S.A.).

### IC_50_ calculation of micronutrients and their synergistic effects on luteolin

The IC_50_ of micronutrients against SARS-CoV-2 3CLpro was measured by varying the concentration of metal chlorides or L-ascorbate at fixed concentrations of 2 μM 3CLpro and 60 µM peptide substrate. The FRET assay was used to measure the inhibition rate of micronutrients against SARS-CoV-2 3CLpro as described here. Upon determining the IC_50_ values for the micronutrients, their synergistic effects on luteolin were assessed by including fixed concentrations of the micronutrients at their IC_50_ values while varying the luteolin concentrations. For example, the concentrations of metal chlorides 3.5 mM Mg^2+^, 0.5 mM Ca^2+^, 0.3 mM Zn^2+^, and 2.3 mM Mn^2+^, as well as 0.3 mM L-ascorbate were maintained fixed in the proteolytic assay. The FRET assay was used to measure the IC_50_ values of luteolin in the presence of different micronutrients were acquired by varying the concentration of luteolin from 3 to 400 µM at fixed concentrations of micronutrients, 2 μM 3CLpro, and 60 µM peptide substrate. Data were fitted as explained earlier and triplicate reactions were analyzed for each data point. The values are displayed as the mean ± standard deviation (SD), *n*=3.

### Differential scanning colorimetry

The thermal stability of 3CLpro in the absence and presence of 100 μM luteolin was assessed by differential scanning calorimetry (DSC) in a Nano-DSC instrument (TA Instruments, New Castle, DE, U.S.A.). The concentration of 3CLpro was 25 μM in buffer containing 20 mM HEPES pH 7.0, 150 mM NaCl, 1 mM EDTA, 1 mM TCEP, and 20% (v/v) DMSO. Thermograms of 3CLpro were collected in the absence and presence of different micronutrients: 0.3 mM ZnCl_2_, 2.3 mM MnCl_2_, 3.5 mM MgCl_2_, 0.5 mM CaCl_2_, or 0.3 mM vitamin C (L-ascorbate). Buffer with and without inhibitor was used as a reference. All samples were scanned from 15°C to 75°C at a temperature ramp rate of 1°C/min. Each sample was scanned twice, with the second scan used as the background reference scan. The melting transitions of all 3CLpro samples were irreversible, as indicated by the lack of signal in the second temperature ramp-up scan. The DSC scans were normalized for protein concentration and baseline corrected by subtracting the reference second scan. The data were converted to plots of excess heat capacity (C_p_) as a function of temperature. The melting point (*T*_m_) was determined from the apex of the thermal transition, and the calorimetric enthalpy (Δ*H*_cal_) was estimated from the area under the thermal transition curve using NanoAnalyze Software v3.11.0 (TA Instruments).

### Molecular docking of peptide substrate and luteolin to 3CLpro

The 3D structure of the SARS-CoV-2 3CLpro amino acid peptide substrate ‘KTSAVLQSGFRKME’ was generated by submitting the peptide sequence to the PEP-FOLD 3 server (https://bioserv.rpbs.univ-paris-diderot.fr/services/PEP-FOLD3/). Next, a crystal structure of 3CLpro (PDB: 7E19) was chosen based on its crystallographic resolution (<2.5 A) and R-value (<0.20), as well as the presence or absence of a crystallographic inhibitor [48,88,89]. The structures of the protein and peptide substrate were then submitted to the HPEPDOCK server (http://huanglab.phys.hust.edu.cn/hpepdock/) for peptide docking, which returned the docking energies for each generated complex [90]. The 10 best 3CLpro-substrate complexes were evaluated for the best peptide binding position in the 3CLpro active site using the PyMOL Molecular Graphics System, Version 3.0 Schrödinger, LLC. 2D interaction maps were generated using LigPlot+ 2.2 [91]. The best 3CLpro-substrate complex was used in the molecular dynamics (MD) analysis.

First, the luteolin 3D structure was submitted to the Marvin Sketch 21.8 program to check for clashes. Luteolin was complexed into SARS-CoV-2 3CLpro (PDB: 7E19) with and without the peptide substrate for docking calculations using MGL Tools 1.5.6 and AutoDock Vina [92–94]. The best affinity energy values for the top nine docking positions of luteolin inside the 3CLpro active site were selected for further analysis. Structural alignment of the crystallographic and docked ligands was performed using the UCSF Chimera program for root mean square deviation (RMSD) calculations [94]. The docking procedure was considered valid when the RMSD between crystallographic and docked ligands was below 2 Å.

### Molecular dynamics (MD) analysis of 3CLpro

The 3CLpro-peptide substrate complex in the absence and presence of luteolin was submitted to 600-ns MD using the GROMACS 2023 program [95]. The CHARMM-GUI tool (https://www.charmm-gui.org/) was used to parameterize and generate GROMACS files of 3CLpro and luteolin [96,97]. The 3CLpro structure was loaded into the pdb2mx module, and the structure of luteolin was prepared by adding hydrogens using the Avogadro program and the Perl script for bond parametrizations [98]. Then, the structure of the complex was submitted to the CGenFF tool (https://cgenff.umaryland.edu/) for manual adjustments [99]. The following steps were performed for the MD simulations: (a) solvation with a dodecahedron spc216 water box with a distance of 1.0 nm from the complex; (b) system neutralization by adding four sodium ions using a maximum of 50000 steps of a Verlet 1.0 Coulomb type cutoff; (c) energy minimization of a maximum of 50,000 steps of a particle mesh Ewald (PME) Coulomb type; (d) ligand restriction of the non-hydrogen atoms from the ligand by index generation; (e) a temperature equilibration step of 1 ns under a constant number of particles (N), system volume (V) and temperature (T) (NVT); (f) a pressure equilibration step of 1 ns under constant N, pressure (P) and T (NPT); and (g) 600 ns of MD simulations. Graphs were obtained of the RMSD of the 3CLpro-peptide substrate complex in the absence and presence of luteolin.

### Interactome analysis

Interactions between SARS-CoV-2 and *Homo sapiens* proteins were taken from the BIOGRID database [100,101]. Since the STRING database has a more standardized scoring system, it was used to identify high-confidence interactions (>900) between NSP5/3CLpro and human proteins [102,103]. A chemical–protein interaction network of luteolin and *H. sapiens* proteins was also obtained from the STITCH database using the criterion of high-confidence interactions (>700) [104]. These three interactomes were merged in Cytoscape version 3.7.2 using the Merge under Network tool [105].

The central proteins in the system (hub, bottleneck, and hub-bottleneck) were identified by applying the betweenness and degree centrality with the betweenness and degree functions of the igraph package [106]. Cluster analysis was also performed in an R environment using the fast-greedy community function [106,107]. Thus, the focus was the cluster of proteins related to 3CLPro, luteolin, ascorbate, Zn^2+^, and Mg^2+^. In these clusters, only the first-degree interactions of 3CLPro with central proteins, luteolin, Zn^2+^, Mg^2+^, and vitamin C were highlighted. Functional enrichment analysis of these groups of proteins was performed to identify biological processes related to central proteins that interact with 3CLpro and small molecules. For this, the BINGO plugin from the Cytoscape program was used with the hypergeometric test applying the Benjamini & Hochberg false discovery rate (FDR) correction test for multiple comparisons [108].

## Supplementary Material

Supplementary videos

## Data Availability

The authors declare that all data that support the findings of this study are available within the files associated with this paper.

## Abbreviations

ARDS: acute respiratory distress syndrome
ACE2: angiotensin-converting enzyme II
3CLpro: 3-chymotrypsin-like protease
COVID-19: coronavirus disease 2019
CPE: cytopathic effect
DMSO: dimethyl sulfoxide
DSC: differential scanning calorimetry
EBV: Epstein-Barr virus
FDA: Food and Drug Administration
FDR: false discovery rate
FRET: fluorescence resonance energy transfer
HDAC2: histone deacetylase 2
MD: molecular dynamics
MERS: Middle East Respiratory Syndrome
MMP: matrix metalloproteinase
nsp: nonstructural proteins
PLpro: papain-like protease
PRMT5: protein arginine methyltransferase 5
RB1: retinoblastoma protein
RMSD: root-mean-square deviation

## References

[1] Jiang C., Yao X., Zhao Y., Wu J., Huang P., Pan C. et al. (2020) Comparative review of respiratory diseases caused by coronaviruses and influenza A viruses during epidemic season. Microbes Infect. 22, 236–244 10.1016/j.micinf.2020.05.005 32405236 PMC7217786

[2] Schoeman D. and Fielding B.C. (2019) Coronavirus envelope protein: current knowledge. Virol J. 16, 69 10.1186/s12985-019-1182-0 31133031 PMC6537279

[3] Zhou P., Yang X.L., Wang X.G., Hu B., Zhang L., Zhang W. et al. (2020) A pneumonia outbreak associated with a new coronavirus of probable bat origin. Nature 579, 270–273 10.1038/s41586-020-2012-7 32015507 PMC7095418

[4] Malone B., Urakova N., Snijder E.J. and Campbell E.A. (2022) Structures and functions of coronavirus replication-transcription complexes and their relevance for SARS-CoV-2 drug design. Nat. Rev. Mol. Cell Biol. 23, 21–39 10.1038/s41580-021-00432-z 34824452 PMC8613731

[5] Saksena N., Bonam S.R. and Miranda-Saksena M. (2021) Epigenetic lens to visualize the severe acute respiratory syndrome coronavirus-2 (SARS-CoV-2) infection in COVID-19 pandemic. Front Genet. 12, 581726–581726 10.3389/fgene.2021.581726 33828579 PMC8019793

[6] Pillaiyar T., Manickam M., Namasivayam V., Hayashi Y. and Jung S.H. (2016) An overview of severe acute respiratory syndrome-coronavirus (SARS-CoV) 3CL protease inhibitors: peptidomimetics and small molecule chemotherapy. J. Med. Chem. 59, 6595–6628 10.1021/acs.jmedchem.5b01461 26878082 PMC7075650

[7] Liang P.H. (2006) Characterization and inhibition of SARS-coronavirus main protease. Curr. Top. Med. Chem. 6, 361–376 10.2174/156802606776287090 16611148

[8] Rut W., Lv Z., Zmudzinski M., Patchett S., Nayak D., Snipas S.J. et al. (2020) Activity profiling and crystal structures of inhibitor-bound SARS-CoV-2 papain-like protease: A framework for anti-COVID-19 drug design. Sci. Adv. 6, 42 eabd4596 10.1126/sciadv.abd4596 33067239 PMC7567588

[9] Kurt Yilmaz N., Swanstrom R. and Schiffer C.A. (2016) Improving viral protease inhibitors to counter drug resistance. Trends Microbiol. 24, 547–557 10.1016/j.tim.2016.03.010 27090931 PMC4912444

[10] Baez-Santos Y.M., St John S.E. and Mesecar A.D. (2015) The SARS-coronavirus papain-like protease: Structure, function and inhibition by designed antiviral compounds. Antivir. Res. 115, 21–38 10.1016/j.antiviral.2014.12.015 25554382 PMC5896749

[11] Gao X., Qin B., Chen P., Zhu K., Hou P., Wojdyla J.A. et al. (2020) Crystal structure of SARS-CoV-2 papain-like protease. Acta Pharm. Sin. B. 11, 237–245 10.1016/j.apsb.2020.08.014 32895623 PMC7467110

[12] Al Adem K., Ferreira J.C., Fadl S. and Rabeh W.M. (2022) pH profiles of 3-chymotrypsin-like protease (3CLpro) from SARS-CoV-2 elucidate its catalytic mechanism and a histidine residue critical for activity. J. Biol. Chem. 299 2 102790 10.1016/j.jbc.2022.102790 36509143 PMC9733303

[13] Ferreira J.C., Fadl S., Villanueva A.J. and Rabeh W.M. (2021) Catalytic dyad residues His41 and Cys145 impact the catalytic activity and overall conformational fold of the main SARS-CoV-2 protease 3-chymotrypsin-like protease. Front. Chem. 9, 692168 10.3389/fchem.2021.692168 34249864 PMC8264439

[14] Maurya V.K., Kumar S., Bhatt M.L.B. and Saxena S.K. (2020) Therapeutic development and drugs for the treatment of COVID-19. Coronavirus Dis. 2019 (COVID-19) 109–126 10.1007/978-981-15-4814-7_10

[15] Saksena N. (2020) Current and future challenges in drug therapeutics for Sars-Cov-2 infection in Covid-19 pandemic. Am. J. Biomed. Res. 9, 153–157 10.34297/AJBSR.2020.09.001374

[16] Alexander J., Tinkov A., Strand T.A., Alehagen U., Skalny A. and Aaseth J. (2020) Early nutritional interventions with zinc, selenium and vitamin D for raising anti-viral resistance against progressive COVID-19. Nutrients 12, 2358 10.3390/nu12082358 32784601 PMC7468884

[17] Chambial S., Dwivedi S., Shukla K.K., John P.J. and Sharma P. (2013) Vitamin C in disease prevention and cure: an overview. Indian J. Clin. Biochem. 28, 314–328 10.1007/s12291-013-0375-3 24426232 PMC3783921

[18] Hamada A.M. (2020) Vitamins, omega-3, magnesium, manganese, and thyme can boost our immunity and protect against COVID-19. Eur. J. Biol. Res. 10, 271–295

[19] Name J.J., Souza A.C.R., Vasconcelos A.R., Prado P.S. and Pereira C.P.M. (2020) Zinc, Vitamin D and Vitamin C: Perspectives for COVID-19 with a focus on physical tissue barrier integrity. Front Nutr. 7, 606398 10.3389/fnut.2020.606398 33365326 PMC7750357

[20] Rondanelli M., Miccono A., Lamburghini S., Avanzato I., Riva A., Allegrini P. et al. (2018) Self-Care for Common Colds: The Pivotal Role of Vitamin D, Vitamin C, Zinc, and Echinacea in Three Main Immune Interactive Clusters (Physical Barriers, Innate and Adaptive Immunity) Involved during an Episode of Common Colds-Practical Advice on Dosages and on the Time to Take These Nutrients/Botanicals in order to Prevent or Treat Common Colds. Evid Based Complement Alternat Med. 2018, 5813095 10.1155/2018/5813095 29853961 PMC5949172

[21] Wintergerst E.S., Maggini S. and Hornig D.H. (2006) Immune-enhancing role of vitamin C and zinc and effect on clinical conditions. Ann. Nutr. Metab. 50, 85–94 10.1159/000090495 16373990

[22] Andreini C., Arnesano F. and Rosato A. (2022) The zinc proteome of SARS-CoV-2. Metallomics 14, 7 mfac047 10.1093/mtomcs/mfac047 35767875 PMC9314716

[23] Arentz S., Hunter J., Yang G., Goldenberg J., Beardsley J., Myers S.P. et al. (2020) Zinc for the prevention and treatment of SARS-CoV-2 and other acute viral respiratory infections: a rapid review. Adv. Integr. Med. 7, 252–260 10.1016/j.aimed.2020.07.009 32837895 PMC7395818

[24] Barocas J.A., So-Armah K., Cheng D.M., Lioznov D., Baum M., Gallagher K. et al. (2019) Zinc deficiency and advanced liver fibrosis among HIV and hepatitis C co-infected anti-retroviral naïve persons with alcohol use in Russia. PloS ONE 14, e0218852–e0218852 10.1371/journal.pone.0218852 31246992 PMC6597160

[25] Kumar A., Kubota Y., Chernov M. and Kasuya H. (2020) Potential role of zinc supplementation in prophylaxis and treatment of COVID-19. MedHypotheses 144, 109848 10.1016/j.mehy.2020.109848 PMC7247509 32512490

[26] Pal A., Squitti R., Picozza M., Pawar A., Rongioletti M., Dutta A.K. et al. (2021) Zinc and COVID-19: Basis of Current Clinical Trials. Biol. Trace Elem. Res. 199, 2882–2892 10.1007/s12011-020-02437-9 33094446 PMC7580816

[27] Read S.A., Obeid S., Ahlenstiel C. and Ahlenstiel G. (2019) The role of zinc in antiviral immunity. Adv. Nutr. 10, 696–710 10.1093/advances/nmz013 31305906 PMC6628855

[28] Skalny A.V., Rink L., Ajsuvakova O.P., Aschner M., Gritsenko V.A., Alekseenko S.I. et al. (2020) Zinc and respiratory tract infections: Perspectives for COVID-19 (Review). Int. J. Mol. Med. 46, 17–26 10.3892/ijmm.2020.4575 32319538 PMC7255455

[29] Skrajnowska D. and Bobrowska-Korczak B. (2019) Role of zinc in immune system and anti-cancer defense mechanisms. Nutrients 11, 2273 10.3390/nu11102273 31546724 PMC6835436

[30] te Velthuis A.J.W., van den Worm S.H.E., Sims A.C., Baric R.S., Snijder E.J. and van Hemert M.J. (2010) Zn2+ inhibits coronavirus and arterivirus RNA polymerase activity in vitro and zinc ionophores block the replication of these viruses in cell culture. PLoS Pathog. 6, e1001176–e1001176 10.1371/journal.ppat.1001176 21079686 PMC2973827

[31] Jothimani D., Kailasam E., Danielraj S., Nallathambi B., Ramachandran H., Sekar P. et al. (2020) COVID-19: Poor outcomes in patients with zinc deficiency. Int. J. Infect. Dis. 100, 343–349 10.1016/j.ijid.2020.09.014 32920234 PMC7482607

[32] Rani I., Goyal A., Bhatnagar M., Manhas S., Goel P., Pal A. et al. (2021) Potential molecular mechanisms of zinc- and copper-mediated antiviral activity on COVID-19. Nutr. Res. 92, 109–128 10.1016/j.nutres.2021.05.008 34284268 PMC8200255

[33] Shetler C.L., Ferreira J.C., Cardoso T.H.S., Silva E.M.A., Saksena N.K. and Rabeh W.M. (2022) Therapeutic potential of metal ions for COVID-19: insights from the papain-like protease of SARS-CoV-2. Biochem. J. 479, 2175–2193 10.1042/BCJ20220380 36205308

[34] Nabi-Afjadi M., Karami H., Goudarzi K., Alipourfard I. and Bahreini E. (2021) The effect of vitamin D, magnesium and zinc supplements on interferon signaling pathways and their relationship to control SARS-CoV-2 infection. Clin Mol Allergy 19, 21 10.1186/s12948-021-00161-w 34749737 PMC8573303

[35] Yu R., Chen L., Lan R., Shen R. and Li P. (2020) Computational screening of antagonists against the SARS-CoV-2 (COVID-19) coronavirus by molecular docking. Int. J. Antimicrobial Agents 56, 106012 10.1016/j.ijantimicag.2020.106012 PMC7205718 32389723

[36] Artese A., Svicher V., Costa G., Salpini R., Di Maio V.C., Alkhatib M. et al. (2020) Current status of antivirals and druggable targets of SARS CoV-2 and other human pathogenic coronaviruses. Drug Resist. Updat. 53, 100721 10.1016/j.drup.2020.100721 33132205 PMC7448791

[37] Maffucci I. and Contini A. (2020) In silico drug repurposing for SARS-CoV-2 main proteinase and spike proteins. J. Proteome Res. 19, 4637–4648 10.1021/acs.jproteome.0c00383 32893632 PMC7640956

[38] Kumar D., Chandel V., Raj S. and Rathi B. (2020) In silico identification of potent FDA approved drugs against Coronavirus COVID-19 main protease: A drug repurposing approach. 7, 10

[39] Adhikari N., Amin S.A. and Jha T. (2021) Dissecting the Drug Development Strategies Against SARS-CoV-2 Through Diverse Computational Modeling Techniques. In In Silico Modeling of Drugs Against Coronaviruses: Computational Tools and Protocols(Roy K., ed.), pp. 329–431, Springer US, New York, NY

[40] Shawan M., Halder S.K. and Hasan M.A. (2021) Luteolin and abyssinone II as potential inhibitors of SARS-CoV-2: an in silico molecular modeling approach in battling the COVID-19 outbreak. Bull Natl. Res. Cent. 45, 27 10.1186/s42269-020-00479-6 33495684 PMC7816153

[41] Molavi Z., Razi S., Mirmotalebisohi S.A., Adibi A., Sameni M., Karami F. et al. (2021) Identification of FDA approved drugs against SARS-CoV-2 RNA dependent RNA polymerase (RdRp) and 3-chymotrypsin-like protease (3CLpro), drug repurposing approach. Biomed. Pharmacother. 138, 111544 10.1016/j.biopha.2021.111544 34311539 PMC8011644

[42] Ferreira J.C., Fadl S., Ilter M., Pekel H., Rezgui R., Sensoy O. et al. (2021) Dimethyl sulfoxide reduces the stability but enhances catalytic activity of the main SARS-CoV-2 protease 3CLpro. FASEB J. 35, 8 e21774 10.1096/fj.202100994 34324734 PMC8441638

[43] Jin Z., Zhao Y., Sun Y., Zhang B., Wang H., Wu Y. et al. (2020) Structural basis for the inhibition of SARS-CoV-2 main protease by antineoplastic drug carmofur. Nat. Struct. Mol. Biol. 27, 529–532 10.1038/s41594-020-0440-6 32382072

[44] Ma C., Hu Y., Townsend J.A., Lagarias P.I., Marty M.T., Kolocouris A. et al. (2020) Ebselen, disulfiram, carmofur, PX-12, tideglusib, and shikonin are nonspecific promiscuous SARS-CoV-2 main protease inhibitors. ACS Pharmacol. Transl. Sci. 3, 1265–1277 10.1021/acsptsci.0c00130 33330841 PMC7571300

[45] Ma C., Sacco M.D., Hurst B., Townsend J.A., Hu Y., Szeto T. et al. (2020) Boceprevir, GC-376, and calpain inhibitors II, XII inhibit SARS-CoV-2 viral replication by targeting the viral main protease. Cell Res. 30, 678–692 10.1038/s41422-020-0356-z 32541865 PMC7294525

[46] Weglarz-Tomczak E., Tomczak J.M., Talma M., Burda-Grabowska M., Giurg M. and Brul S. (2021) Identification of ebselen and its analogues as potent covalent inhibitors of papain-like protease from SARS-CoV-2. Sci. Rep. 11, 3640 10.1038/s41598-021-83229-6 33574416 PMC7878891

[47] Amporndanai K., Meng X., Shang W., Jin Z., Rogers M., Zhao Y. et al. (2021) Inhibition mechanism of SARS-CoV-2 main protease by ebselen and its derivatives. Nat. Commun. 12, 3061 10.1038/s41467-021-23313-7 34031399 PMC8144557

[48] Konno S., Kobayashi K., Senda M., Funai Y., Seki Y., Tamai I. et al. (2021) 3CL protease inhibitors with an electrophilic arylketone moiety as anti-SARS-CoV-2 agents. J. Med. Chem. 65 4 2926–2939 10.1021/acs.jmedchem.1c00665. 34313428

[49] Ahmad B., Batool M., ul Ain Q., Kim M.S. and Choi S. (2021) Exploring the binding mechanism of PF-07321332 SARS-CoV-2 protease inhibitor through molecular dynamics and binding free energy simulations. Int. J. Mol. Sci. 22, 9124, ARTN 10.3390/ijms22179124 34502033 PMC8430524

[50] Nunes R.R., Costa M.D., Santos B.D., Fonseca A.L., Ferreira L.S., Chagas R.C. et al. (2016) Successful application of virtual screening and molecular dynamics simulations against antimalarial molecular targets. Mem. Inst. Oswaldo Cruz. 111, 721–730 10.1590/0074-02760160207 27982302 PMC5146734

[51] Westermaier Y., Barril X. and Scapozza L. (2015) Virtual screening: an in silico tool for interlacing the chemical universe with the proteome. Methods 71, 44–57 10.1016/j.ymeth.2014.08.001 25193260

[52] Seelinger G., Merfort I. and Schempp C.M. (2008) Anti-oxidant, anti-inflammatory and anti-allergic activities of luteolin. Planta Med. 74, 1667–1677 10.1055/s-0028-1088314 18937165

[53] Du A., Zheng R., Disoma C., Li S., Chen Z., Li S. et al. (2021) Epigallocatechin-3-gallate, an active ingredient of Traditional Chinese Medicines, inhibits the 3CLpro activity of SARS-CoV-2. Int. J. Bio. Macromo. 176, 1–12 10.1016/j.ijbiomac.2021.02.012 PMC7859723 33548314

[54] Du R., Cooper L., Chen Z., Lee H., Rong L. and Cui Q. (2021) Discovery of chebulagic acid and punicalagin as novel allosteric inhibitors of SARS-CoV-2 3CL(pro). Antiviral Res. 190, 105075 10.1016/j.antiviral.2021.105075 33872675 PMC8052511

[55] Chen L., Li J., Luo C., Liu H., Xu W., Chen G. et al. (2006) Binding interaction of quercetin-3-beta-galactoside and its synthetic derivatives with SARS-CoV 3CL(pro): structure-activity relationship studies reveal salient pharmacophore features. Bioorg. Med. Chem. 14, 8295–8306 10.1016/j.bmc.2006.09.014 17046271 PMC7125754

[56] Ryu Y.B., Jeong H.J., Kim J.H., Kim Y.M., Park J.-Y., Kim D. et al. (2010) Biflavonoids from Torreya nucifera displaying SARS-CoV 3CLpro inhibition. Bioorg. Med. Chem. 18, 7940–7947 10.1016/j.bmc.2010.09.035 20934345 PMC7126309

[57] Ahmadian R., Rahimi R. and Bahramsoltani R. (2020) Kaempferol: an encouraging flavonoid for COVID-19. Bol. Latinoam. Caribe Plantas Med. Aromat. 19, 492–494 10.37360/blacpma.20.19.5.33

[58] Sadati S.M., Gheibi N., Ranjbar S. and Hashemzadeh M.S. (2019) Docking study of flavonoid derivatives as potent inhibitors of influenza H1N1 virus neuraminidase. Biomed Rep. 10, 33–38 30588301 10.3892/br.2018.1173 PMC6299203

[59] Su H., Yao S., Zhao W., Li M., Liu J., Shang W. et al. (2020.04.13) Discovery of baicalin and baicalein as novel, natural product inhibitors of SARS-CoV-2 3CL protease in vitro.bioRxiv 038687 10.1101/2020.04.13.038687

[60] Mucsi I., Gyulai Z. and Beladi I. (1992) Combined effects of flavonoids and acyclovir against herpesviruses in cell cultures. Acta Microbiol. Hung. 39, 137–147 1339152

[61] Mehla R., Bivalkar-Mehla S. and Chauhan A. (2011) A flavonoid, luteolin, cripples HIV-1 by abrogation of tat function. PloS ONE 6, e27915 10.1371/journal.pone.0027915 22140483 PMC3227592

[62] Kang O.-H., Choi J.-G., Lee J.-H. and Kwon D.-Y. (2010) Luteolin isolated from the flowers of Lonicera japonica suppresses inflammatory mediator release by blocking NF-kappaB and MAPKs activation pathways in HMC-1 cells. Molecules 15, 385–398 10.3390/molecules15010385 20110898 PMC6257122

[63] Williamson G. and Clifford M.N. (2010) Colonic metabolites of berry polyphenols: the missing link to biological activity? Br. J. Nutr. 104, S48–S66 10.1017/S0007114510003946 20955650

[64] Xu L., Su W.H., Jin J., Chen J.W., Li X.J., Zhang X.Y. et al. (2014) Identification of luteolin as enterovirus 71 and coxsackievirus A16 inhibitors through reporter viruses and cell viability-based screening. Viruses-Basel 6, 2778–2795 10.3390/v6072778 PMC4113793 25036464

[65] Kim H.P., Son K.H., Chang H.W. and Kang S.S. (2004) Anti-inflammatory plant flavonoids and cellular action mechanisms. J. Pharmacol. Sci. 96, 229–245 10.1254/jphs.CRJ04003X 15539763

[66] Fan W., Qian S., Qian P. and Li X. (2016) Antiviral activity of luteolin against Japanese encephalitis virus. Virus Res. 220, 112–116 10.1016/j.virusres.2016.04.021 27126774

[67] Knipping K., Garssen J. and van't Land B. (2012) An evaluation of the inhibitory effects against rotavirus infection of edible plant extracts. Virol J. 9, 137 10.1186/1743-422X-9-137 22834653 PMC3439294

[68] Murali K.S., Sivasubramanian S., Vincent S., Murugan S.B., Giridaran B., Dinesh S. et al. (2015) Anti-chikungunya activity of luteolin and apigenin rich fraction from Cynodon dactylon. Asian Pac. J. Trop. Med. 8, 352–358 10.1016/S1995-7645(14)60343-6 26003593

[69] Borbone N., Piccialli G., Roviello G.N. and Oliviero G. (2021) Nucleoside analogs and nucleoside precursors as drugs in the fight against SARS-CoV-2 and other coronaviruses. Molecules 26, 986 10.3390/molecules26040986 33668428 PMC7918729

[70] Al Adem K., Ferreira J.C., Fadl S., Mustafa M. and Rabeh W.M. (2023) Key allosteric and active site residues of SARS-CoV-2 3CLpro are promising drug targets. Biochem. J. 480 11 791–813 10.1042/BCJ20230027 37254750

[71] Pavlova A., Lynch D.L., Daidone I., Zanetti-Polzi L., Smith M.D., Chipot C. et al. (2021) Inhibitor binding influences the protonation states of histidines in SARS-CoV-2 main protease. Chem. Sci. 12, 1513–1527 10.1039/D0SC04942E 35356437 PMC8899719

[72] Gordon D.E., Jang G.M., Bouhaddou M., Xu J., Obernier K., White K.M. et al. (2020) A SARS-CoV-2 protein interaction map reveals targets for drug repurposing. Nature 583, 459–468 10.1038/s41586-020-2286-9 32353859 PMC7431030

[73] Barnes P.J. (2009) Role of HDAC2 in the pathophysiology of COPD. Annu. Rev. Physiol. 71, 451–464 10.1146/annurev.physiol.010908.163257 18817512

[74] Xu P., Ye S., Li K., Huang M., Wang Q., Zeng S. et al. (2019) NOS1 inhibits the interferon response of cancer cells by S-nitrosylation of HDAC2. J. Exp. Clin. Cancer Res. 38, 483 10.1186/s13046-019-1448-9 31805977 PMC6896289

[75] Kim H. and Ronai Z.A. (2020) PRMT5 function and targeting in cancer. Cell Stress 4, 199–215 10.15698/cst2020.08.228 32743345 PMC7380451

[76] Cai T., Yu Z., Wang Z., Liang C. and Richard S. (2021) Arginine methylation of SARS-Cov-2 nucleocapsid protein regulates RNA binding, its ability to suppress stress granule formation, and viral replication. J. Biol. Chem. 297, 100821 10.1016/j.jbc.2021.100821 34029587 PMC8141346

[77] Bhardwaj K., Liu P., Leibowitz J.L. and Kao C.C. (2012) The coronavirus endoribonuclease Nsp15 interacts with retinoblastoma tumor suppressor protein. J. Virol. 86, 4294–4304 10.1128/JVI.07012-11 22301153 PMC3318636

[78] Nagesh P.T. and Husain M. (2016) Influenza A virus dysregulates host histone deacetylase 1 that inhibits viral infection in lung epithelial cells. J. Virol. 90, 4614–4625 10.1128/JVI.00126-16 26912629 PMC4836332

[79] He B. and Garmire L. (2020) Prediction of repurposed drugs for treating lung injury in COVID-19. F1000Res 9, 609 10.12688/f1000research.23996.2 32934806 PMC7468567

[80] Bouhaddou M., Memon D., Meyer B., White K.M., Rezelj V.V., Correa Marrero M. et al. (2020) The global phosphorylation landscape of SARS-CoV-2 infection. Cell 182, 685–712, e619 10.1016/j.cell.2020.06.034 32645325 PMC7321036

[81] Baier A., Nazaruk J., Galicka A. and Szyszka R. (2018) Inhibitory influence of natural flavonoids on human protein kinase CK2 isoforms: effect of the regulatory subunit. Mol. Cell. Biochem. 444, 35–42 10.1007/s11010-017-3228-1 29188536 PMC6002439

[82] Yao X., Jiang W., Yu D. and Yan Z. (2019) Luteolin inhibits proliferation and induces apoptosis of human melanoma cells in vivo and in vitro by suppressing MMP-2 and MMP-9 through the PI3K/AKT pathway. Food Funct. 10, 703–712 10.1039/C8FO02013B 30663726

[83] C D.A.-M., Couto A.E.S., Campos L.C.B., Vasconcelos T.F., Michelon-Barbosa J., Corsi C.A.C. et al. (2021) MMP-2 and MMP-9 levels in plasma are altered and associated with mortality in COVID-19 patients. Biomed. Pharmacother. 142, 112067 10.1016/j.biopha.2021.112067 34449310 PMC8376652

[84] Ferreira J.C. and Rabeh W.M. (2020) Biochemical and biophysical characterization of the main protease, 3-chymotrypsin-like protease (3CLpro) from the novel coronavirus SARS-CoV 2. Sci. Rep. 10, 22200 10.1038/s41598-020-79357-0 33335206 PMC7747600

[85] Xue X., Yang H., Shen W., Zhao Q., Li J., Yang K. et al. (2007) Production of authentic SARS-CoV M(pro) with enhanced activity: application as a novel tag-cleavage endopeptidase for protein overproduction. J. Mol. Biol. 366, 965–975 10.1016/j.jmb.2006.11.073 17189639 PMC7094453

[86] Kao R.Y., To A.P., Ng L.W., Tsui W.H., Lee T.S., Tsoi H.W. et al. (2004) Characterization of SARS-CoV main protease and identification of biologically active small molecule inhibitors using a continuous fluorescence-based assay. FEBS Lett. 576, 325–330 10.1016/j.febslet.2004.09.026 15498556 PMC7134596

[87] Tea F., Ospina Stella A., Aggarwal A., Ross Darley D., Pilli D., Vitale D. et al. (2021) SARS-CoV-2 neutralizing antibodies: Longevity, breadth, and evasion by emerging viral variants. PLoS Med. 18, e1003656 10.1371/journal.pmed.1003656 34228725 PMC8291755

[88] Kleywegt G.J. and Brunger A.T. (1996) Checking your imagination: applications of the free R value. Structure 4, 897–904 10.1016/S0969-2126(96)00097-4 8805582

[89] Karplus P.A. and Diederichs K. (2012) Linking crystallographic model and data quality. Science 336, 1030–1033 10.1126/science.1218231 22628654 PMC3457925

[90] Zhou P., Jin B., Li H. and Huang S.Y. (2018) HPEPDOCK: a web server for blind peptide-protein docking based on a hierarchical algorithm. Nucleic Acids Res. 46, W443–W450 10.1093/nar/gky357 29746661 PMC6030929

[91] Laskowski R.A. and Swindells M.B. (2011) LigPlot+: multiple ligand-protein interaction diagrams for drug discovery. J. Chem. Inf. Model. 51, 2778–2786 10.1021/ci200227u 21919503

[92] Morris G.M., Huey R., Lindstrom W., Sanner M.F., Belew R.K., Goodsell D.S. et al. (2009) AutoDock4 and AutoDockTools4: Automated docking with selective receptor flexibility. J. Comput. Chem. 30, 2785–2791 10.1002/jcc.21256 19399780 PMC2760638

[93] Trott O. and Olson A.J. (2010) AutoDock Vina: improving the speed and accuracy of docking with a new scoring function, efficient optimization, and multithreading. J. Comput. Chem. 31, 455–461 10.1002/jcc.21334 19499576 PMC3041641

[94] Pettersen E.F., Goddard T.D., Huang C.C., Couch G.S., Greenblatt D.M., Meng E.C. et al. (2004) UCSF Chimera–a visualization system for exploratory research and analysis. J. Comput. Chem. 25, 1605–1612 10.1002/jcc.20084 15264254

[95] Abraham M.J., Murtola T., Schulz R., Páll S., Smith J.C., Hess B. et al. (2015) GROMACS: High performance molecular simulations through multi-level parallelism from laptops to supercomputers. SoftwareX 1-2, 19–25 10.1016/j.softx.2015.06.001

[96] Jo S., Kim T., Iyer V.G. and Im W. (2008) CHARMM-GUI: a web-based graphical user interface for CHARMM. J. Comput. Chem. 29, 1859–1865 10.1002/jcc.20945 18351591

[97] Justin A.L. (2018) From proteins to perturbed hamiltonians: a suite of tutorials for the GROMACS-2018 molecular simulation package [Article v1.0]. Living J. Computational Mol. Sci. 1 1 5068

[98] Hanwell M.D., Curtis D.E., Lonie D.C., Vandermeersch T., Zurek E. and Hutchison G.R. (2012) Avogadro: an advanced semantic chemical editor, visualization, and analysis platform. J. Cheminformatics 4, 17 10.1186/1758-2946-4-17 PMC3542060 22889332

[99] Vanommeslaeghe K., Hatcher E., Acharya C., Kundu S., Zhong S., Shim J. et al. (2010) CHARMM general force field: a force field for drug-like molecules compatible with the CHARMM all-atom additive biological force fields. J. Comput. Chem. 31, 671–690 10.1002/jcc.21367 19575467 PMC2888302

[100] Chatr-Aryamontri A., Oughtred R., Boucher L., Rust J., Chang C., Kolas N.K. et al. (2017) The BioGRID interaction database: 2017 update. Nucleic. Acids. Res. 45, D369–D379 10.1093/nar/gkw1102 27980099 PMC5210573

[101] Hermjakob H., Montecchi-Palazzi L., Bader G., Wojcik J., Salwinski L., Ceol A. et al. (2004) The HUPO PSI's molecular interaction format–a community standard for the representation of protein interaction data. Nat. Biotechnol. 22, 177–183 10.1038/nbt926 14755292

[102] Szklarczyk D., Gable A.L., Lyon D., Junge A., Wyder S., Huerta-Cepas J. et al. (2019) STRING v11: protein-protein association networks with increased coverage, supporting functional discovery in genome-wide experimental datasets. Nucleic Acids Res. 47, D607–D613 10.1093/nar/gky1131 30476243 PMC6323986

[103] Szklarczyk D., Gable A.L., Nastou K.C., Lyon D., Kirsch R., Pyysalo S. et al. (2021) The STRING database in 2021: customizable protein-protein networks, and functional characterization of user-uploaded gene/measurement sets. Nucleic Acids Res. 49, D605–D612 10.1093/nar/gkaa1074 33237311 PMC7779004

[104] Szklarczyk D., Santos A., von Mering C., Jensen L.J., Bork P. and Kuhn M. (2016) STITCH 5: augmenting protein-chemical interaction networks with tissue and affinity data. Nucleic Acids Res. 44, D380–D384 10.1093/nar/gkv1277 26590256 PMC4702904

[105] Shannon P., Markiel A., Ozier O., Baliga N.S., Wang J.T., Ramage D. et al. (2003) Cytoscape: a software environment for integrated models of biomolecular interaction networks. Genome Res. 13, 2498–2504 10.1101/gr.1239303 14597658 PMC403769

[106] Csárdi G. and Nepusz T. (2006) The igraph software package for complex network research. Int. J. Complex Syst. 1695

[107] Clauset A., Newman M.E.J. and Moore C. (2004) Finding community structure in very large networks. Phys. Rev. E. 70, 066111 10.1103/PhysRevE.70.066111 15697438

[108] Maere S., Heymans K. and Kuiper M. (2005) BiNGO: a Cytoscape plugin to assess overrepresentation of Gene Ontology categories in Biological Networks. Bioinformatics 21, 3448–3449 10.1093/bioinformatics/bti551 15972284

